# Cell-to-cell interaction analysis of prognostic ligand-receptor pairs in human pancreatic ductal adenocarcinoma

**DOI:** 10.1016/j.bbrep.2021.101126

**Published:** 2021-09-04

**Authors:** Sayaka R. Suzuki, Akihiro Kuno, Haruka Ozaki

**Affiliations:** a Program in Human Biology, School of Integrative and Global Majors, University of Tsukuba, 1-1-1 Tennodai, Tsukuba, Ibaraki, 305-8575, Japan; b Bioinformatics Laboratory, Faculty of Medicine, University of Tsukuba, Tsukuba 1-1-1 Tennodai, Tsukuba, Ibaraki, 305-8577, Japan; c Department of Anatomy and Embryology, Faculty of Medicine, University of Tsukuba, Tsukuba 1-1-1 Tennodai, Tsukuba, Ibaraki, 305-8577, Japan; d Center for Artificial Intelligence Research, University of Tsukuba, 1-1-1 Tennodai, Tsukuba, Ibaraki, 305-8577, Japan

**Keywords:** Cell-cell communication, Ligand-receptor pairs, scRNA-seq, Survival analysis, Pancreatic ductal adenocarcinoma

## Abstract

Cell-to-cell interactions (CCIs) through ligand-receptor (LR) pairs in the tumor microenvironment underlie the poor prognosis of pancreatic ductal adenocarcinoma (PDAC). However, there is scant knowledge of the association of CCIs with PDAC prognosis, which is critical to the identification of potential therapeutic candidates. Here, we sought to identify the LR pairs associated with PDAC patient prognosis by integrating survival analysis and single-cell CCI prediction. Via survival analysis using gene expression from cancer cohorts, we found 199 prognostic LR pairs. CCI prediction based on single-cell RNA-seq data revealed the enriched LR pairs associated with poor prognosis. Notably, the CCIs involved epithelial tumor cells, cancer-associated fibroblasts, and tumor-associated macrophages through integrin-related and *ANXA1*–*FPR* pairs. Finally, we determined that CCIs involving 33 poor-prognostic LR pairs were associated with tumor grade. Although the clinical implication of the set of LR pairs must be determined, our results may provide potential therapeutic targets in PDAC.

## Highlights

• We found 199 prognostic ligand-receptor pairs of pancreatic ductal adenocarcinoma.
• PDAC scRNA-seq data showed cell-cell interactions for the prognostic LR pairs.
• We determined that CCIs involving 33 LR pairs were associated with tumor grade.

## 1 Introduction

Pancreatic ductal adenocarcinoma (PDAC), the most common type of pancreatic cancer, is diagnosed as a high-risk malignant neoplasm with a 5-year survival rate less than 10% [1,2]. Although conventional therapies such as surgery and chemoradiation target PDAC itself, recent studies suggest that its poor prognosis is tightly associated with cell-to-cell interactions (CCIs) through ligand-receptor (LR) pairs in the tumor microenvironment [3]. The most recent and reliable cancer therapies targeting CCIs are immune checkpoint inhibitors against cytotoxic T-lymphocyte-associated protein 4 (CTLA-4) and programmed cell death protein 1 (PD-1) or programmed cell death ligand 1 (PD-L1) by activating T cells to eliminate tumor cells [4]. However, these immunotherapies have limited clinical benefits in PDAC [5], suggesting that further and comprehensive research into the CCIs associated with PDAC prognosis is critical to the identification of potential therapeutic candidates.

Single-cell RNA sequencing (scRNA-seq) can reveal the CCIs in the tumor microenvironment [6,7]. Indeed, scRNA-seq recently revealed the cell-type compositions of the tumor microenvironment in PDAC patients [8]. However, further light needs to be shed on the CCIs in PDAC.

Here, we used RNA-seq to extract LR pairs associated with patient prognosis from a publicly available cohort dataset. Next, we investigated the CCIs involving the prognostic LR pairs using scRNA-seq data from PDAC patients [8]. The results showed that CCIs of epithelial tumor cells, cancer-associated fibroblasts, and tumor-associated macrophages through integrin-related and *ANXA1*–*FPR* pairs were poor-prognostic CCIs and are potential therapeutic targets.

## 2 Materials and Methods

### 2.1 Survival analysis of the cancer patients

**Data acquisition** To evaluate the association between LR pairs and patient prognosis, we obtained the RNA-seq data of PDAC from three different cohorts in the International Cancer Genome Consortium (ICGC) repository: PAAD-US (n = 132), PACA-AU (n = 75), and PACA-CA (n = 149). Most of the PDAC RNA-seq data of the ICGC were derived from primary tumors (345 of 356; Table S1). To annotate tumor grades and stages, we used ICGC's Specimen Data File for PACA-AU and PACA-CA projects, whereas we downloaded the clinical information from the NIH GDC Data Portal for PAAD-US. All of the RNA-seq data were normalized by CPM (counts per million). Then, we extracted 2,187 LR pairs contained in the connectomeDB2020 database [9].

**Patient stratification** For each LR pair, a patient was designated “high” if the sum of the gene expressions of the LR pairs was equal to or greater than the median of the sum of the gene expressions of the other pairs. Otherwise, the expression of an LR pair was designated “low”.

**Survival analysis** The overall survival rate of the cancer patients was used for survival analysis with the “survival” package (version 3.2.7) in R (version 3.6.3). Statistical significance was evaluated by the Peto and Peto modification of the Gehan-Wilcoxon test. The hazard ratio (HR) was calculated by the exponentiated coefficients of the Cox regression model. Kaplan-Meier plots were drawn using the “ggsurvplot” function in the “survminer” package (version 0.4.8). We performed survival analysis for each cohort and combined the p-values from the three cohorts by Edgington's method using the “sump” function in the “metap” package (version 1.4). Lastly, Storey's method for multiple testing correction [10] was used for multiple testing correction using the “qvalue” package (version 2.18.0).

**LR pairs associated with prognosis** The LR pairs associated with patient prognosis were determined as follows: (1) a Storey's *q*-value < 0.1 and (2) an HR > 1 (or HR < 1) in all cohorts.

### 2.2 Gene enrichment analysis

We used Enrichr to perform gene enrichment analysis of the LR pairs associated with patient prognosis [11].

### 2.3 Preprocessing of scRNA-seq data

We used the scRNA-seq data of tumors from 10 PDAC patients originally reported by Lin et al. [8]. The data were downloaded from the Gene Expression Omnibus with the accession number GSE154778 and had been generated by Chromium v2 (10x Genomics) and HiSeq 4000 (Illumina). We used the data of primary tumors from 10 patients in this study, which comprised 8,000 cells and 32,738 genes. The cell-type annotation was kindly provided by the authors of the original study [8].

### 2.4 Cell-cell interaction analysis

We used NATMI (commit value: ac7fca2) [9,12] to estimate cell-cell communications between cell-type pairs mediated by LR pairs. Specifically, NATMI ExtractEdges.py was applied to the scRNA-seq data from each patient via Python 3.6.5. The mean expression weight, which is the product of the mean ligand expression level in a cell type and the mean receptor expression level in a cell type, was used for the downstream analysis.

For each cell-type pair, adjusted enrichment of poor- and good-prognostic LR pairs in the detected CCIs was calculated. A patient-wise adjusted enrichment was defined as $m_{p}/n_{p}\cdot N/M$ where $m_{p}$ and $n_{p}$ are the numbers of poor- or good-prognostic LR pairs and all LR pairs detected for a patient p, respectively. M represents the number of poor- or good-prognostic LR pairs, and N represents all LR pairs. The mean of patient-wise adjusted enrichments across patients was then defined as the adjusted enrichment.

To screen CCIs related to a high mean expression weight at pathological grade 4 in the patients, we calculated the quartiles of the weights of all LR pairs detected in grade 4. We then selected LR pairs with weights above the third quartile.

### 2.5 Data visualization

Data visualization was performed using R (4.0.5). Heatmaps were drawn using the “ComplexHeatmap” package (version 2.6.2) [13]. Network diagrams were visualized using the “qgraph” package (version 1.6.9).

## 3 Results

### 3.1 Screening of LR pairs associated with PDAC patient survival

**Fig. 1:**
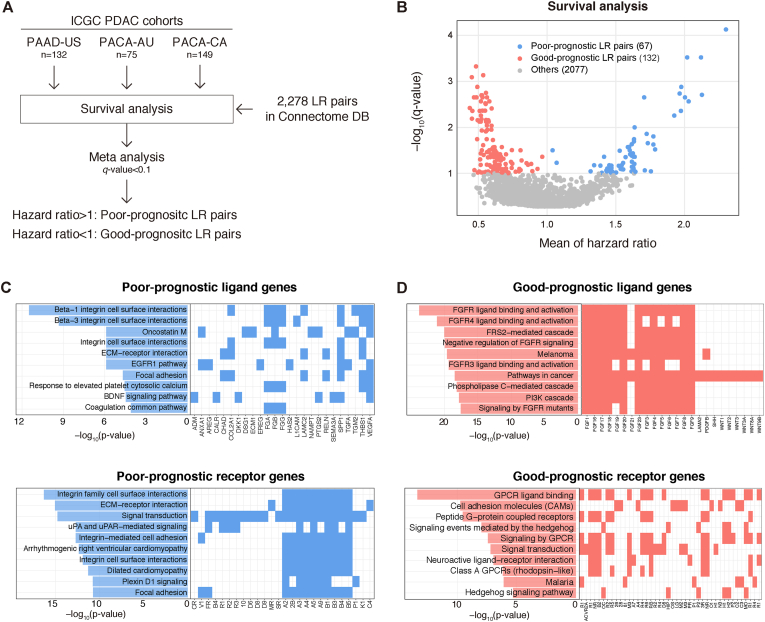
Survival analysis of LR pairs associated with PDAC prognosis (A) Schema of the survival analysis. LR pair: ligand-receptor pair. (B) The x-axis represents the mean hazard ratio while the y-axis represents the −log_10_(*q*-value) by Storey's method. Blue, red, and gray points represent poor-prognostic LR pairs (HR > 1), good-prognostic LR pairs (HR < 1), and the others, respectively. (C) Gene enrichment analysis of ligand and receptor genes of poor-prognostic LR pairs using Enrichr. For each of the enriched gene sets in the BioPlanet 2019 database, the −log_10_(*p*-value) and overlapping genes are shown. (D) Gene enrichment analysis of ligand and receptor genes of good-prognostic LR pairs using Enrichr. (For interpretation of the references to colour in this figure legend, the reader is referred to the Web version of this article.)

To screen LR interactions associated with PDAC prognosis, we used RNA-seq data from PDAC patients whose outcomes are available across three cohorts (TCGA [n = 132], Canadian [n = 149], and Australian [n = 74] cohorts in the ICGC database). Then, we performed survival analysis (Fig. 1A and Materials and Methods). We targeted 2,187 pairs of ligand and receptor genes provided by the connectomeDB2020 database [9]. From the screening, we found that 199 LR pairs were significantly associated with patient prognosis (67 pairs as poor-prognostic pairs and 132 pairs as good-prognostic pairs) (Fig. 1B, **S1**, **S2**, and Table S2). The screened LR pairs included genes that have been associated with PDAC patient prognosis. For example, the screened LR pair with the highest HR was *SEMA4B*–*DCBLD2* (*SEMA4B* is a ligand for *DCBLD2*). All three cohorts showed poor prognosis in a reproducible manner (Fig. S1). Previous work demonstrated that the *SEMA4B* and *DCBLD2* interaction is involved in the regulation of the motility of lung cancer cell lines [14]. In addition, a recent study using PDAC cohorts reported that *DCBLD2* was associated with poor survival in PDAC [15].

Next, to characterize the screened LR pairs, we performed enrichment pathway analysis. For the poor-prognostic ligand and receptor genes, the integrin-related pathway was highly enriched. For instance, the pathway contained fibrinogen genes, including *FGA*, *FGB*, and *FGG*. Previous studies found that serum fibrinogen levels were associated with a prognostic biomarker for PDAC [16,17], which indicated that fibroblastic stimuli can be transmitted in the PDAC microenvironment. In addition, epidermal growth factor receptors (*EGFR*, *ERBB4*), frizzled receptors (*FZD6*, *FZD8*, *FZD9*, *FZD10*), and formyl peptide receptors (*FPR1*, *FPR2*, *FPR3*) were enriched in the “signal transduction” term (Fig. 1C). The growth factor receptors and frizzled receptors are well characterized as contributors to PDAC tumor development [18,19]. The formyl peptide receptors are expressed in phagocytes and a recent study showed that the *ANXA1*–*FPRs* pathway promotes PDAC metastasis [20]. On the other hand, the good-prognostic LR pairs were highly enriched in fibroblast growth factor (FGF)-related signaling (Fig. 1D). Although the role of FGFs in PDAC has been controversial [21], a set of good-prognostic LR pairs including *FGF7*, *FGF10*, and *FGFR2* (Table S2) is proposed to exert a tumor suppressive function via reduced oxidative stress and activation of immune surveillance [22,23]. Collectively, these results suggest that the screened LR pairs reflect possible CCIs associated with PDAC patient prognosis.

### 3.2 scRNA-seq reconstructs cell-type pairs underlying cell-cell communication

**Fig. 2:**
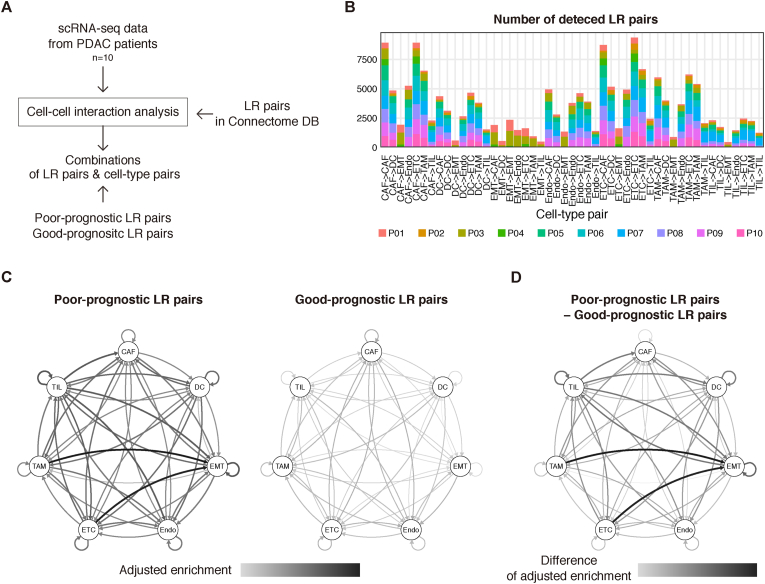
scRNA-seq reconstructs cell-type pairs underlying cell-cell communication (A) Schematic diagram of the scRNA-seq cell-cell communication analysis. (B) Summary of the detected LR pairs per cell-type pair and patient by NATMI. (C) Enrichment of poor- and good-prognostic LR pairs among the detected LR pairs for each cell-type pair. Edge width represents the adjusted enrichment. (D) Difference in the adjusted enrichment between poor- and good-prognostic LR pairs.

As a result of the LR pairs associated with PDAC patient survival, we next sought to identify which cell-type pairs were involved in the CCIs mediated by these LR pairs. Because most of the RNA-seq data used in the survival analysis were derived from primary tumors, we selected published scRNA-seq data from PDAC primary tumors (n = 10) [8] for CCI analysis (Fig. 2A and Table S3). We followed the cell-type annotation in the original study [8], which comprised seven cell-type clusters: CAFs (cancer-associated fibroblasts), DCs (dendritic cells), EMT cells (tumor cells with epithelial-mesenchymal transition characteristics), Endos (endothelial cells), ETCs (epithelial tumor cells), TAMs (tumor-associated macrophages), and TILs (tumor-infiltrating lymphocytes). ETCs comprised the largest proportion in most patients of grades 2 and 3, whereas EMT cells had the highest proportion and CAFs had the second largest proportion in grade 4 (Fig. S3 and Table S4).

We applied NATMI [9] to the scRNA-seq data of each patient to detect active LR pairs between each paired cell type. The amount of LR pairs extracted from NATMI varied among the patients, from 3,575 (P04) to 29,316 (P07) across cell-type pairs (Table S5). This difference was partly due to the variation in the numbers of cells among cell types and patients (Table S6 and Fig. S4). In total cell-type pairs, ETC–ETC contained the most LR pairs detected across patients (total, 9,349), followed by CAF–CAF, CAF–TAM, and ETC–CAF (Fig. 2B and Table S5).

To account for the differences in cell-type composition and in the numbers of LR pairs among patients, we calculated the enrichment of the screened LR pairs relative to all focal LR pairs in NATMI (Materials and Methods). Among the CCIs for all LR pairs tested, the poor-prognostic LR pairs were more enriched than the good-prognostic LR pairs in all cell-type pairs (Fig. 2C and D). Based on this result, we thus focused on the poor-prognostic LR pairs in the subsequent work.

**Fig. 3:**
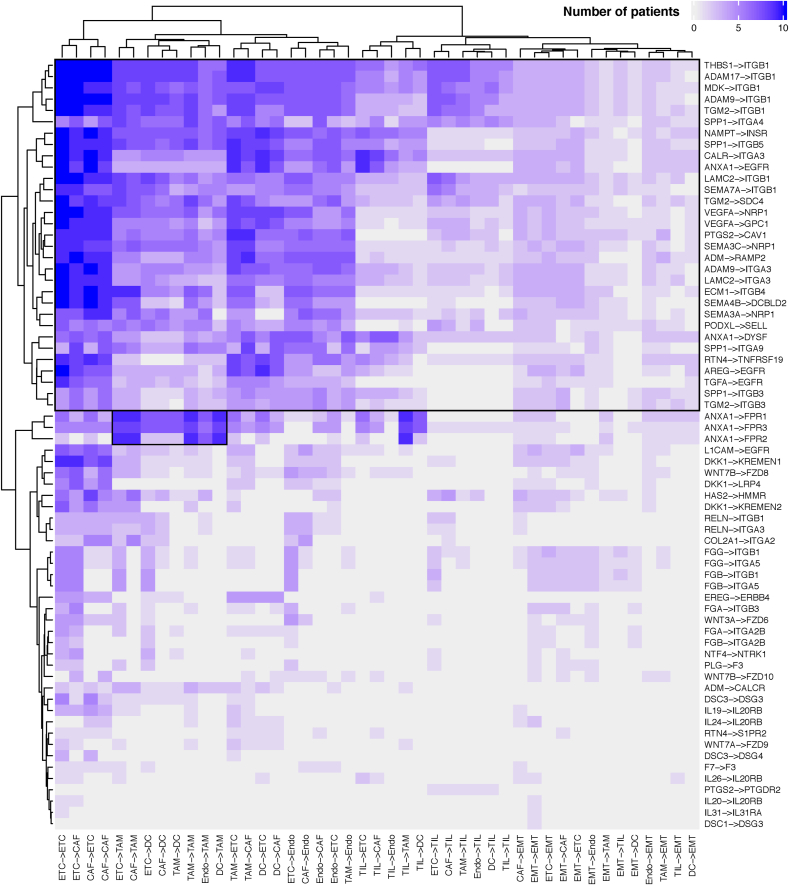
Proportion of cell-cell interactions of poor-prognostic LR pairs in PDAC patients Heatmap of the numbers of patients with a cell-cell interaction (CCI) detected by NATMI. Rectangles indicate the common LR pairs expressed in the PDAC patients.

Next, although the scRNA-seq data revealed the heterogeneity of each patient [8], we hypothesized that the CCIs commonly expressed among the patients might affect PDAC progression. Thus, to uncover the CCIs in each patient, we clustered poor-prognostic LR pairs and cell-type pairs based on the number of detections in all patients. Most of the patients were found to show integrin-related LR pairs through an ETC and CAF interaction and *ANXA1*–*FPR1*/*FPR2*/*FPR3* pairs through an ETC–TAM interaction (Fig. 3). Because PDAC has a strong stromal reaction, mutual communication between ETCs and CAFs is considered to be crucial [24]. Our results could reflect the stromal reaction and uncover the possible LR pairs related to the stromal reaction. On the other hand, our results showed that *ANXA1*–*FPRs* also prevailed in PDAC patients through an ETC–TAM interaction. Previous studies indicated that *ANXA1* sends a signal to *FPRs* and that this signal is related to malignancy in PDAC [20]. However, the cell type receiving the signal was uncharacterized. Our results suggest that TAMs can be a receiver of *ANXA1* signals. Taken together, the results revealed the poor-prognostic CCIs that are commonly displayed in PDAC patients.

### 3.3 Cell-cell communications associated with high-grade PDAC

**Fig. 4:**
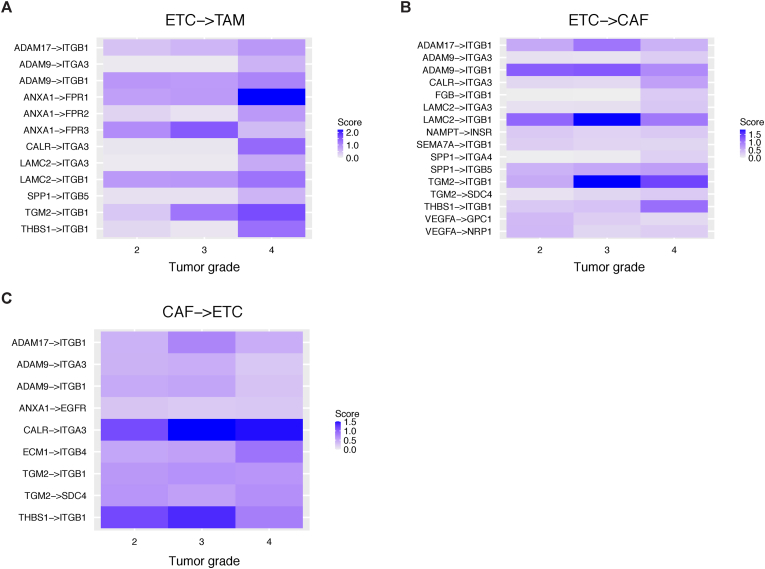
Grade-dependent cell-cell interactions for poor-prognostic LR pairs in PDAC patients Heatmaps of grade-dependent poor-prognostic LR pairs for (A) ETC–TAM, (B) ETC–CAF, and (C) CAF–ETC. Heatmaps for other cell-type pairs are shown in Fig. S5.

Because the different grades reflect the different gene expression profiles [25], we focused on the grade differences in the PDAC patients and investigated grade-dependent CCIs. We further screened the 33 poor-prognostic LR pairs that were highly expressed in grade 4 (Fig. 4 and Fig. S5). We focused on the interactions involving ETCs, CAFs, and TAMs because these CCIs were commonly detected among the 10 PDAC patients. The results revealed that *ANXA1*–*FPR1*/*FPR2* in ETC–TAM showed grade-dependent expression (Fig. 4A). Considering the previous studies suggesting that *ANXA1* expression and TAM infiltration correlate with the pathological grade [26,27], our results indicated that *ANXA1* signals activate TAM infiltration. Furthermore, among the integrin-related CCIs, *CALR*–*ITGA3* on CAF–ETC and ETC–CAF had high expression scores in all grades (Fig. 4B and C). Given recent studies showing that *CALR* promotes epithelial-mesenchymal transition in PDAC [28,29], our results indicate that *CALR* and *ITGA3* could be one of the pathways through which CAFs send the epithelial-mesenchymal transition signal to ETCs. In summary, these results suggest that poor-prognostic CCIs activated along with tumor grades can be potential therapeutic targets for PDAC.

## 4 Discussion

In this study, we screened the LR pairs whose expressions were associated with the survival of PDAC patients and subsequently investigated CCIs mediated by the screened LR pairs based on the scRNA-seq data. We found 67 poor-prognostic LR pairs and 132 good-prognostic LR pairs. The poor-prognostic LR pairs included *ANXA1*–*FPRs* in ETC–TAM and *CALR*–*ITGA3* in CAF–ETC. By combining the survival analysis with the cohort-level bulk RNA-seq data and the cell-cell communication analysis with the scRNA-seq data, our study uncovered the prognostic LR pairs as well as the possible candidate CCIs involving the LR pairs, providing potential therapeutic targets.

The previous studies reported that PDAC contains complex CCIs in the tumor microenvironment that support tumor malignancy [30]. In particular, the integrin signaling of the extracellular matrix and stroma has been described [3]. Our screening suggested that *ITGB3* is one of the most enriched receptors associated with poor prognosis (Fig. 1C). Cilengitide (EMD121974) specifically inhibits integrin αVβ3 and αVβ5 [31], and recent clinical studies indicated its efficacy in various cancers [32]. Our study also revealed the interaction of *ANXA1*–*FPRs* in ETCs and TAMs (Fig. 3, Fig. 4). Previous studies reported that *ANXA1* inhibition reduces tumor growth and immunogenicity activation in breast cancer [33,34]. Because *ANXA1* signaling promotes M2 macrophage polarization in an *FPR2*-dependent manner [35], *ANXA1* inhibition may contribute to PDAC therapy.

There are several limitations to this study. First, we focused on primary tumors throughout this study because most of the PDAC RNA-seq data of the ICGC were derived from primary tumors (345 of 356; Table S1). Further investigation of metastatic PDAC data is needed when the RNA-seq data from metastatic PDAC become more available. Second, we stratified the cohorts based on the sum of expression of the ligand and the receptor genes for each LR pair, but a more appropriate stratification method might exist depending on the distributions of gene expression levels (e.g., binarization for each gene separately). Third, the scRNA-seq data of the same stage or grade showed heterogeneous cell-type compositions (Fig. S3), suggesting that the current sample size might not be sufficient to fully cover the heterogeneity of PDAC. Because the latest research is contributing to ongoing efforts to apply scRNA-seq to multiple cancer types [36], further data acquisition will provide validation cohorts of scRNA-seq data to guarantee the robustness of the current conclusion. In addition, our approach can be adapted to other cancer types with more scRNA-seq data available. Finally, the results of the present study are based on data analyses. Although our approach is beneficial to screen prognostic LR pairs, *in vivo* studies will reinforce our results. Moreover, future experimental validation will ensure the clinical implications of our findings.

In summary, our identified prognostic LR pairs can serve as a potential therapeutic target that complements conventional therapies. Further investigation is required to identify the clinical implications of the LR pairs in PDAC and their association with mutations and oncogenic pathways.

## Declaration of competing interest

The authors declare the following financial interests/personal relationships which may be considered as potential competing interests:

Haruka Ozaki reports financial support was provided by 10.13039/501100001691Japan Society for the Promotion of Science. Akihiro Kuno reports financial support was provided by 10.13039/501100001691Japan Society for the Promotion of Science. Haruka Ozaki reports a relationship with iBioTech that includes: consulting or advisory.

## Availability of data and materials

The codes and datasets are available at the GitHub repository: https://github.com/bioinfo-tsukuba/Suzuki2021_PDAC.

## Appendix A Supplementary data

Supplementary Figures (PDF) Supplementary Figures (PDF)

Supplementary Tables (Excel) Supplementary Tables (Excel)
