# Supplementary material for: Cell-to-cell interaction analysis of prognostic ligand-receptor pairs in human pancreatic ductal adenocarcinoma
Source: Biochem Biophys Rep. 2021 Sep 4;28:101126. doi: 10.1016/j.bbrep.2021.101126 (PMC8426203; doi:10.1016/j.bbrep.2021.101126)
Supplement: Supplementary Figures (PDF) [file mmc1.pdf]

Supplementary Information:

Suzuki *et al*. "Cell-to-cell interaction analysis of prognostic ligand-receptor pairs in human pancreatic ductal adenocarcinoma"

List of Supplementary Figures

Figure S1. Kaplan-Meier plots of poor-prognostic LR pairs.

Figure S2. Kaplan-Meier plots of good prognostic LR pairs.

Figure S3. Cell-type compositions and numbers of cells in the scRNA-seq data.

Figure S4. Relationship of the number of cells with the number of detected LR pairs per cell-type pairs.

Figure S5. Heatmaps of mean expression weights in 33 grade-dependent LR pairs.

List of Suppelementary Tables (provided as Excel spreadsheets)

Table S1. List of PDAC patients with RNA-seq data in the ICGC.

Table S2. List of LR pairs associated with the survival of PDAC patients with RNA-seq data in the ICGC.

Table S3. Patient metadata in the scRNA-seq dataset.

Table S4. scRNA-seq cell-type composition.

Table S5. Number of CCIs detected by NATMI.

Table S6. Comparison of NATMI and cell numbers.

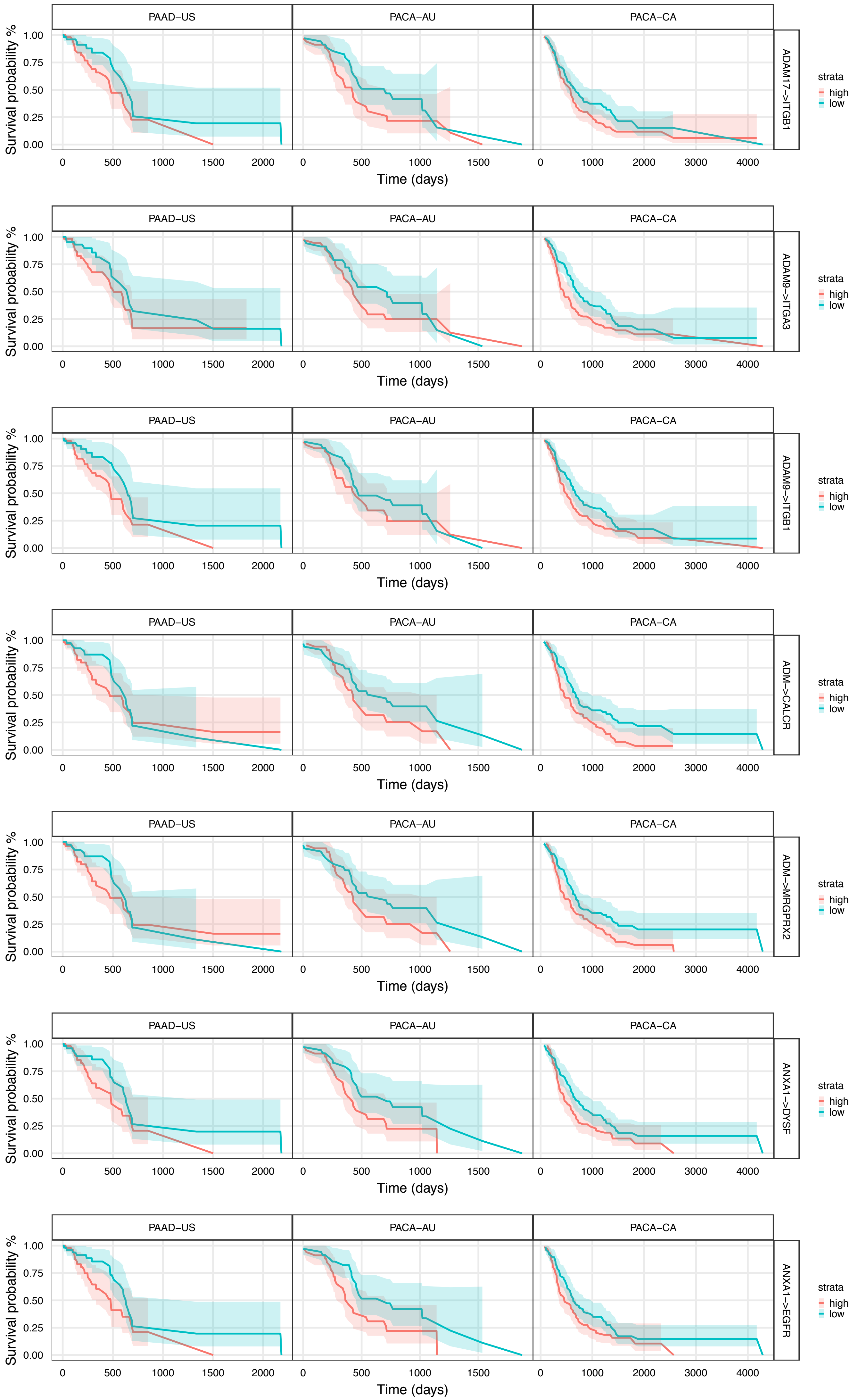
PAAD–US
PACA–AU
PACA–CA
Survival probability %
1.00
0.75
0.50
0.25
0.00
0
500
1000
1500
2000
2000
3000
4000
Time (days)
strata
high
low
ADAM17–>ITGB1
ADAM9–>ITGA3
ADAM9–>ITGB1
ADM–>CALCR
ADM–>MRGPRX2
ANXA1–>DYSF
ANXA1–>EGFR

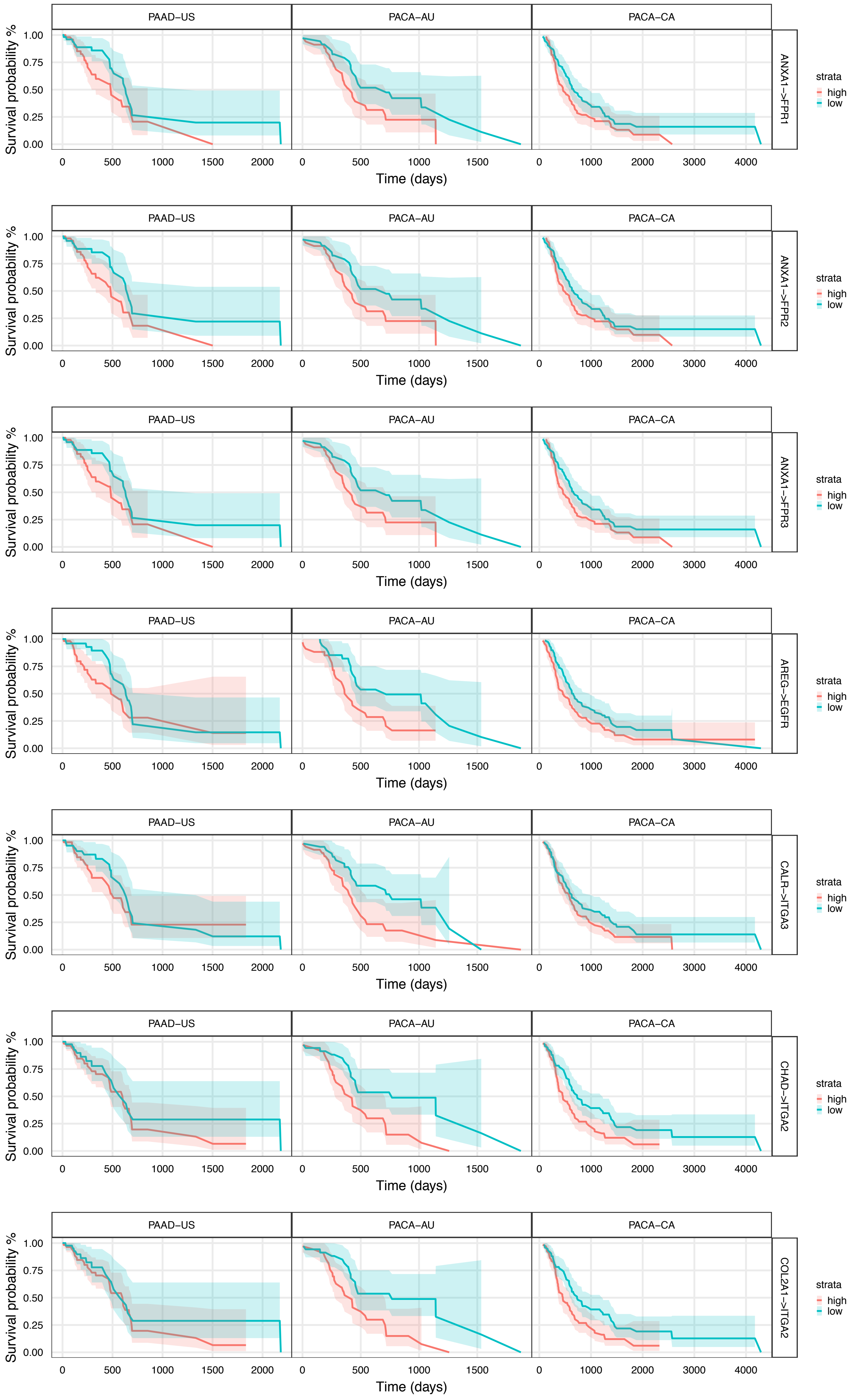
PAAD–US
PACA–AU
PACA–CA
Survival probability %
1.00
0.75
0.50
0.25
0.00
0
500
1000
1500
2000
2000
3000
4000
Time (days)
strata
high
low
ANXA1–>FPR1
ANXA1–>FPR2
ANXA1–>FPR3
AREG–>EGFR
CALR–>ITGA3
CHAD–>ITGA2
COL2A1–>ITGA2

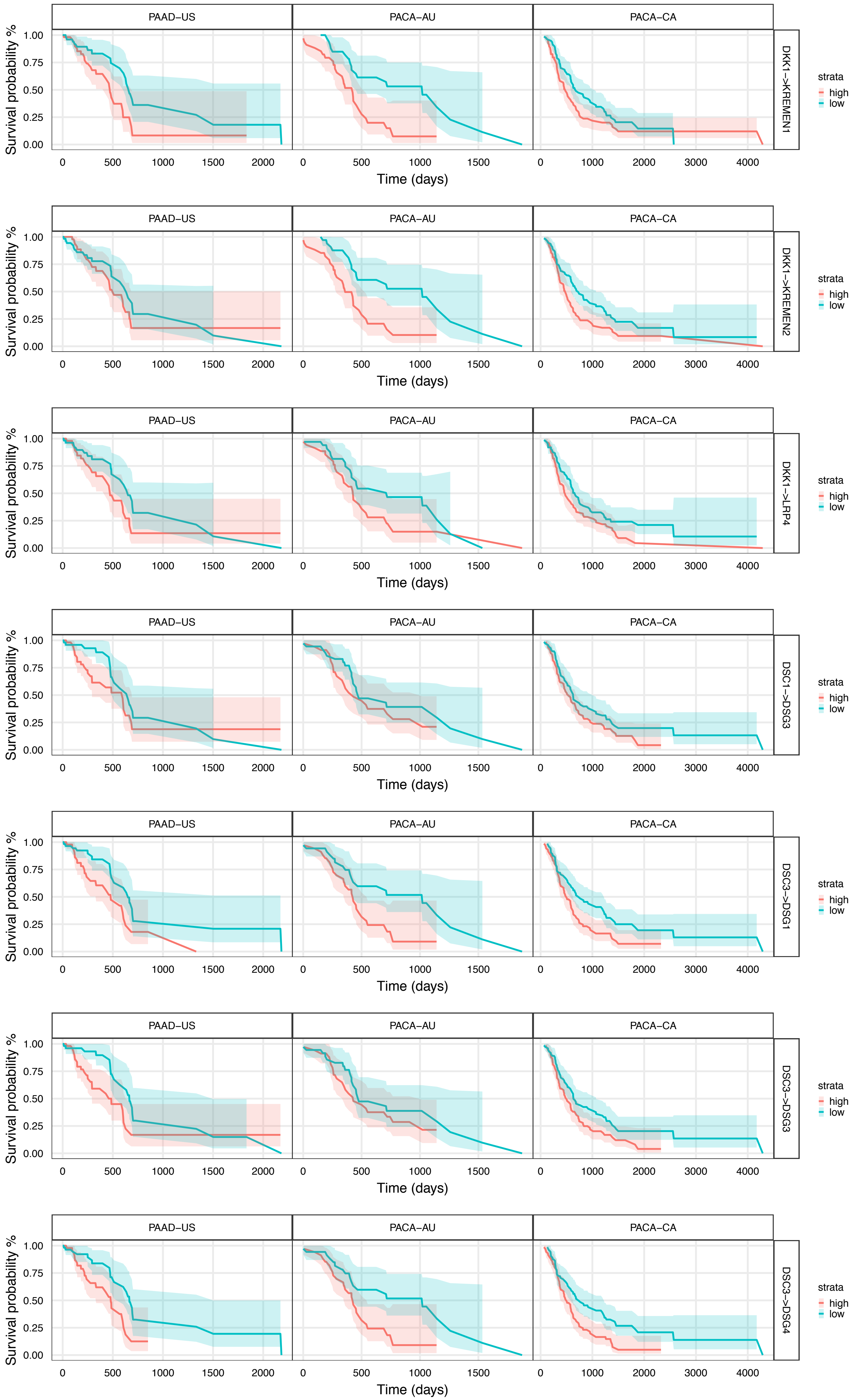
PAAD–US
PACA–AU
PACA–CA
DKK1–>KREMEN1
Survival probability %
1.00
0.75
0.50
0.25
0.00
0
500
1000
1500
2000
2000
3000
4000
Time (days)
strata
high
low
PAAD–US
PACA–AU
PACA–CA
DKK1–>KREMEN2
Survival probability %
Time (days)
strata
high
low
PAAD–US
PACA–AU
PACA–CA
DKK1–>LRP4
Survival probability %
Time (days)
strata
high
low
PAAD–US
PACA–AU
PACA–CA
DSC1–>DSG3
Survival probability %
Time (days)
strata
high
low
PAAD–US
PACA–AU
PACA–CA
DSC3–>DSG1
Survival probability %
Time (days)
strata
high
low
PAAD–US
PACA–AU
PACA–CA
DSC3–>DSG3
Survival probability %
Time (days)
strata
high
low
PAAD–US
PACA–AU
PACA–CA
DSC3–>DSG4
Survival probability %
Time (days)
strata
high
low

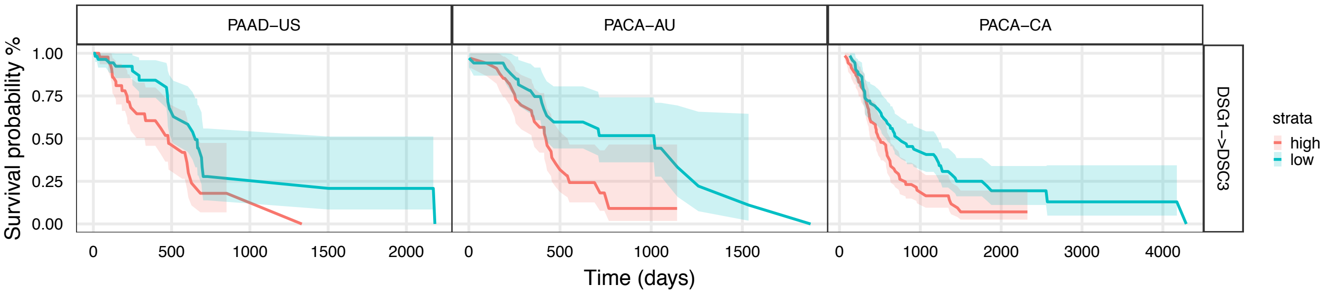
PAAD–US
PACA–AU
PACA–CA
DSG1–>DSC3
Survival probability %
1.00
0.75
0.50
0.25
0.00
0
500
1000
1500
2000
2000
3000
4000
Time (days)
strata
high
low

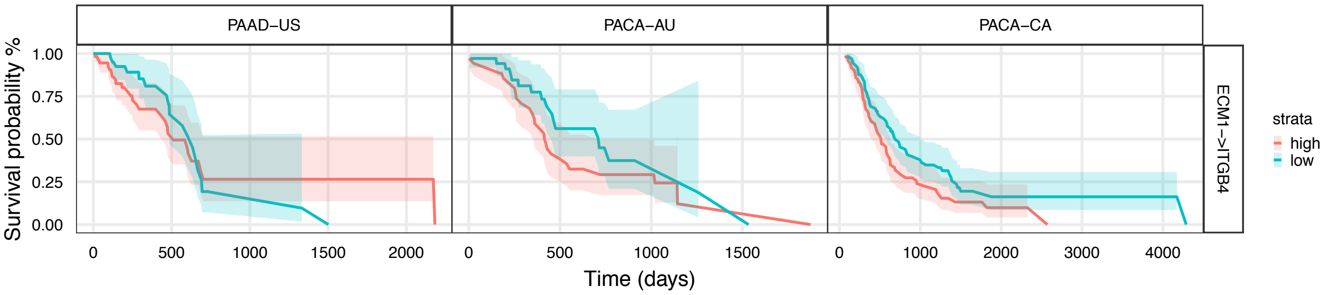
PAAD–US
PACA–AU
PACA–CA
ECM1–>ITGB4
Survival probability %
Time (days)
strata
high
low

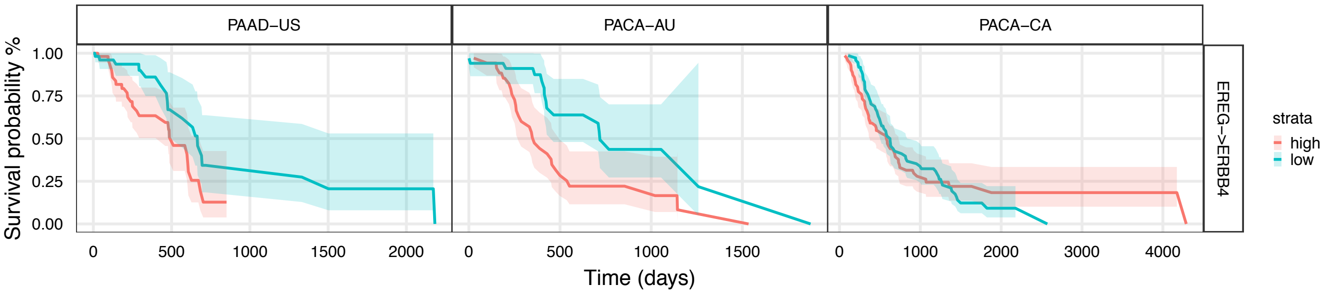
PAAD–US
PACA–AU
PACA–CA
EREG–>ERBB4
Survival probability %
Time (days)
strata
high
low

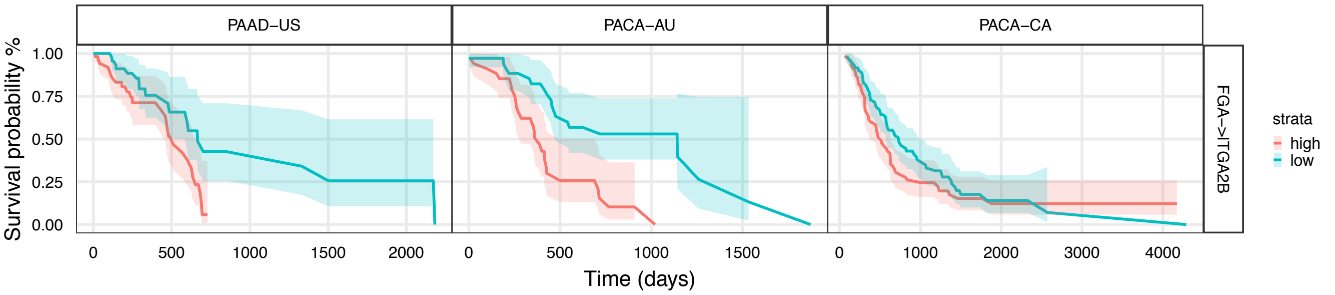
PAAD–US
PACA–AU
PACA–CA
FGA–>ITGA2B
Survival probability %
Time (days)
strata
high
low

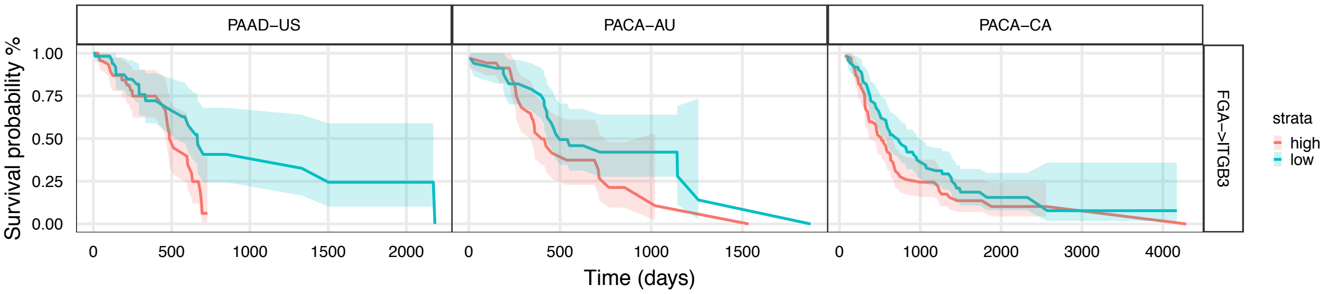
PAAD–US
PACA–AU
PACA–CA
FGA–>ITGB3
Survival probability %
Time (days)
strata
high
low

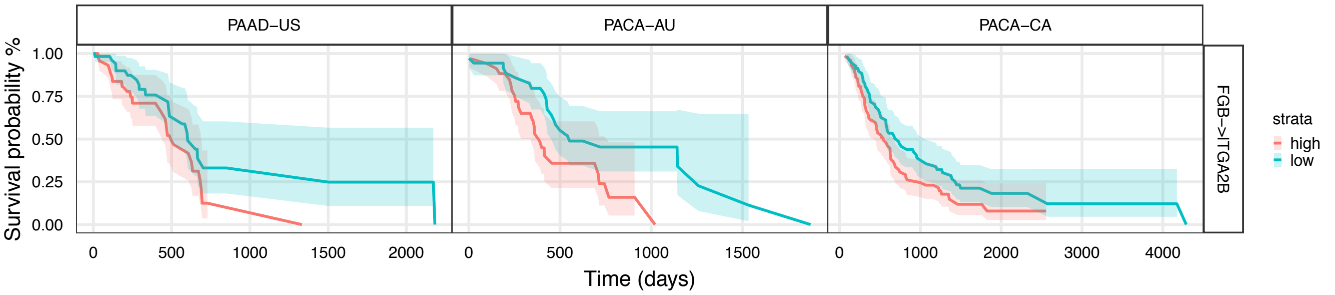
PAAD–US
PACA–AU
PACA–CA
FGB–>ITGA2B
Survival probability %
Time (days)
strata
high
low

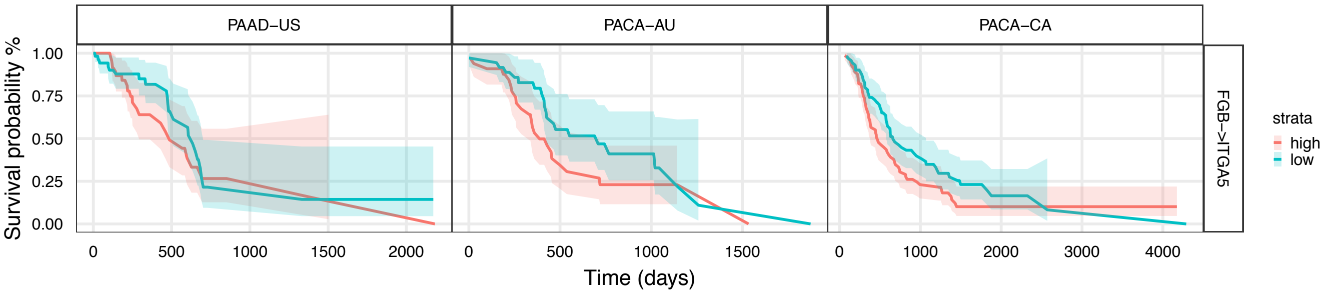
PAAD–US
PACA–AU
PACA–CA
FGB–>ITGA5
Survival probability %
Time (days)
strata
high
low

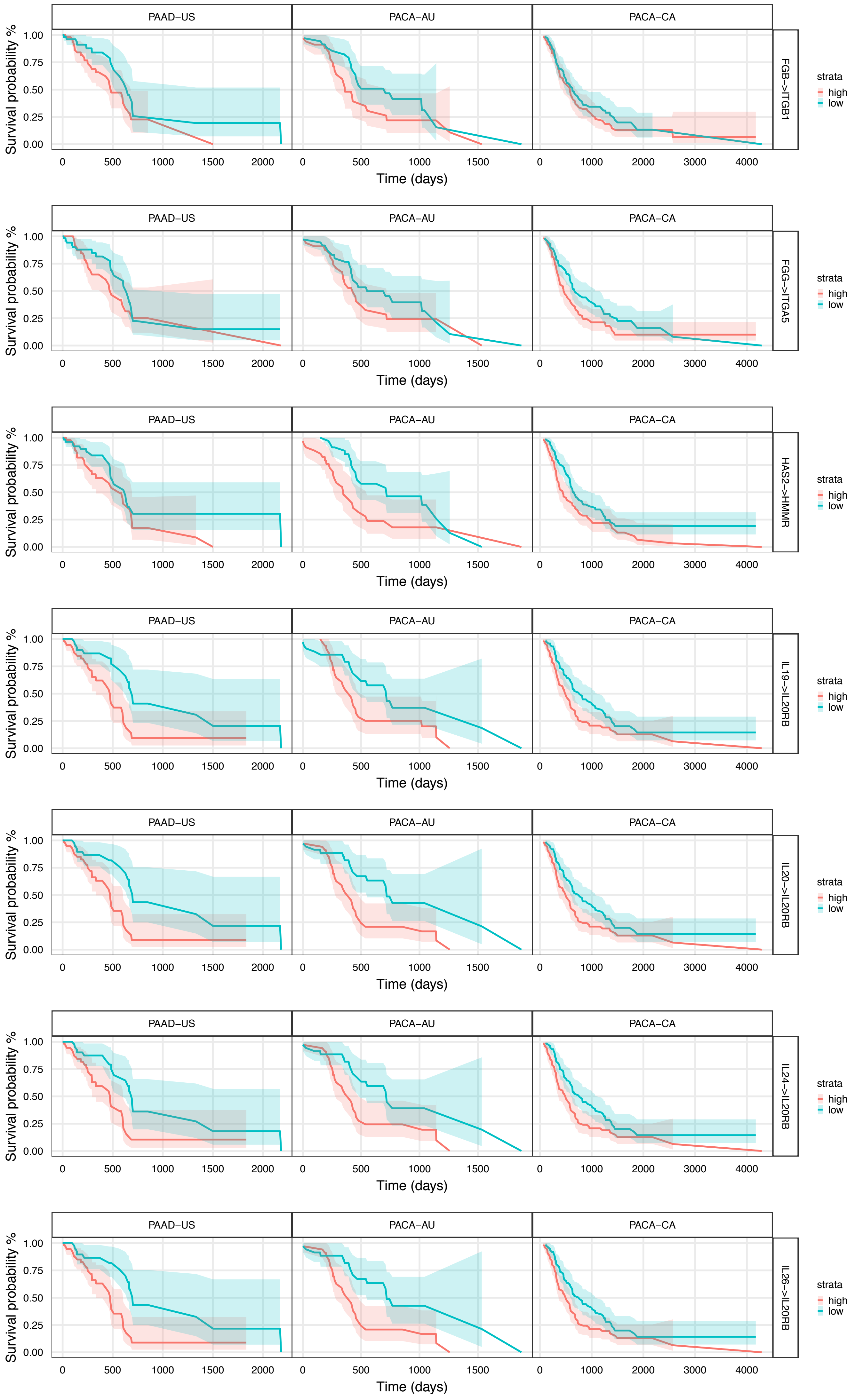
PAAD–US
PACA–AU
PACA–CA
Survival probability %
Time (days)
strata
high
low
FGB–>ITGB1
FGG–>ITGA5
HAS2–>HMMR
IL19–>IL20RB
IL20–>IL20RB
IL24–>IL20RB
IL26–>IL20RB

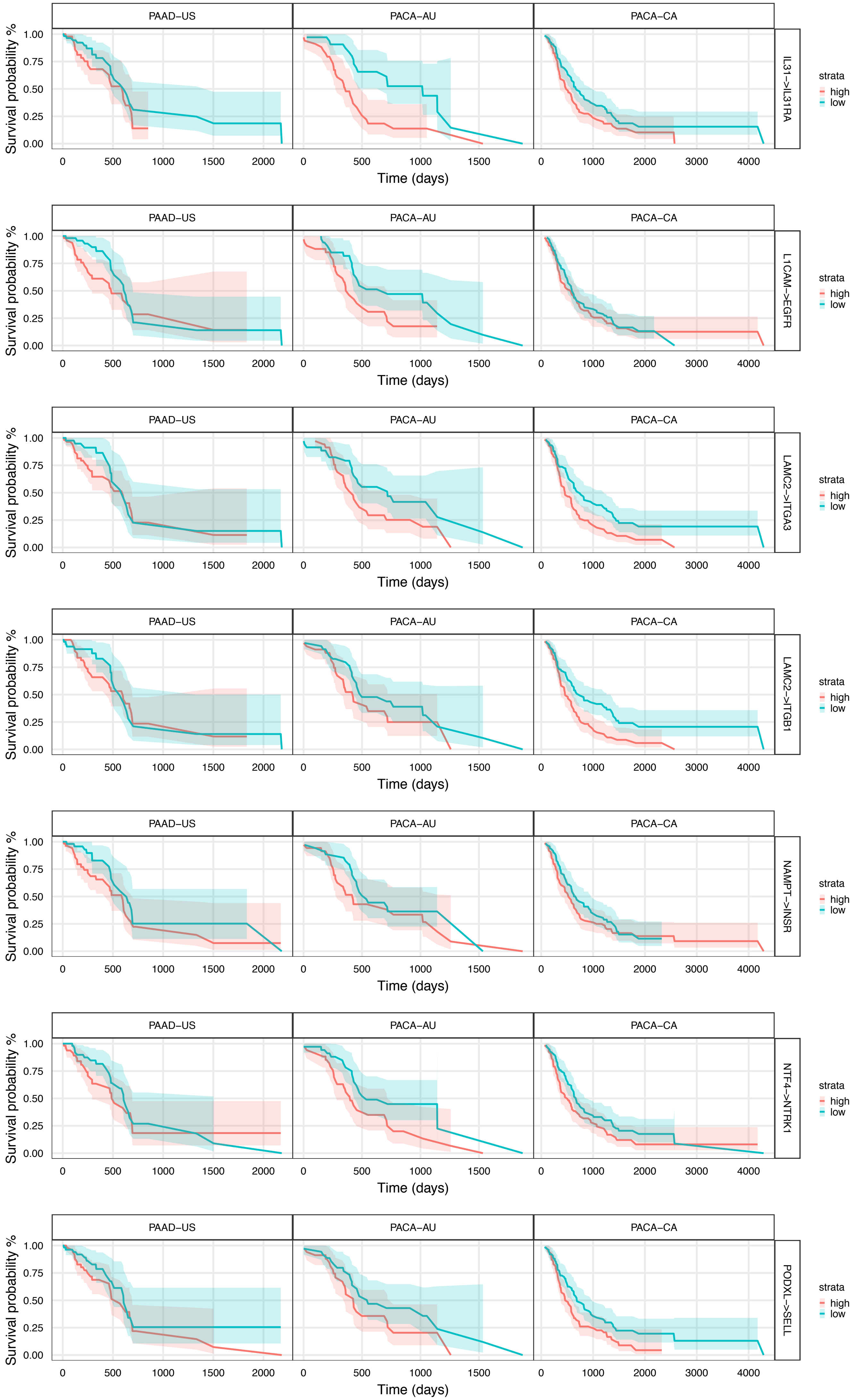
PAAD−US
PACA−AU
PACA−CA
IL31−>IL31RA
L1CAM−>EGFR
LAMC2−>ITGA3
LAMC2−>ITGB1
NAMPT−>INSR
NTF4−>NTRK1
PODXL−>SELL
Survival probability %
Time (days)
strata
high
low
1.00
0.75
0.50
0.25
0.00
0
500
1000
1500
2000
3000
4000

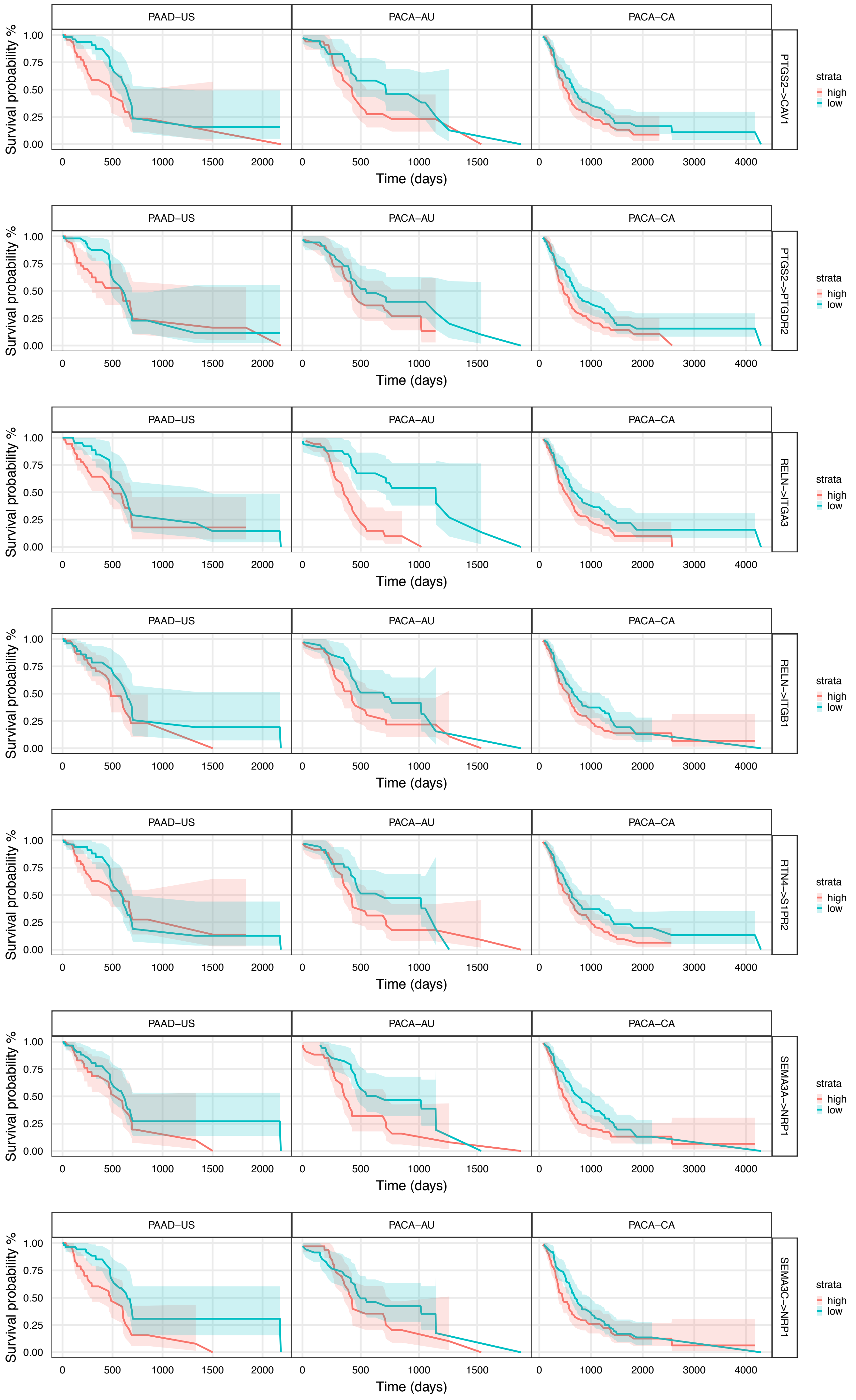

PAAD–US
PACA–AU
PACA–CA
Survival probability %
Time (days)
strata
high
low
PTGS2–>CAV1
PTGS2–>PTGDR2
RELN–>ITGA3
RELN–>ITGB1
RTN4–>S1PR2
SEMA3A–>NRP1
SEMA3C–>NRP1

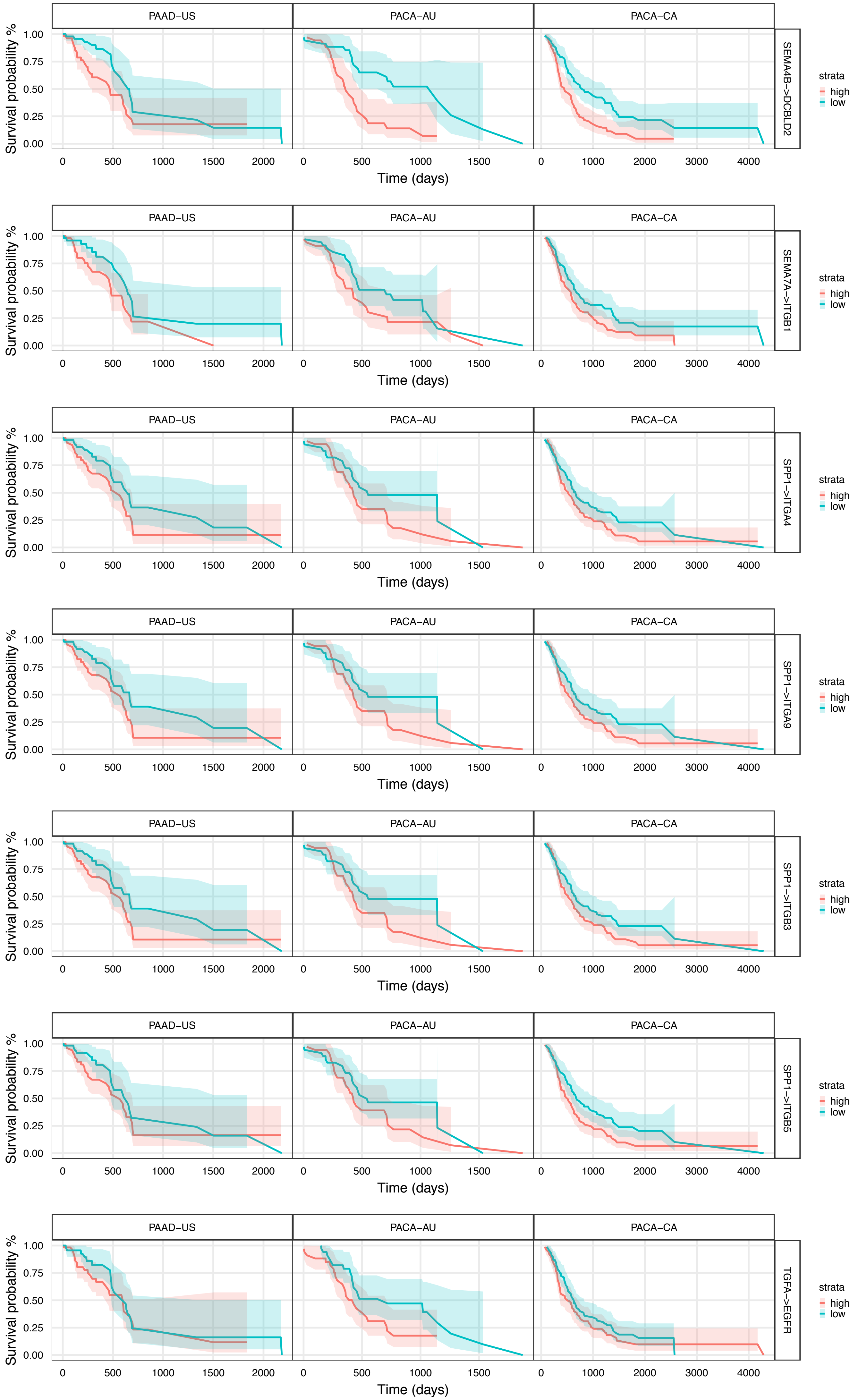
PAAD–US
PACA–AU
PACA–CA
SEMA4B–>DCBLD2
SEMA7A–>ITGB1
SPP1–>ITGA4
SPP1–>ITGA9
SPP1–>ITGB3
SPP1–>ITGB5
TGFA–>EGFR
Survival probability %
1.00
0.75
0.50
0.25
0.00
Time (days)
0
500
1000
1500
2000
3000
4000
strata
high
low

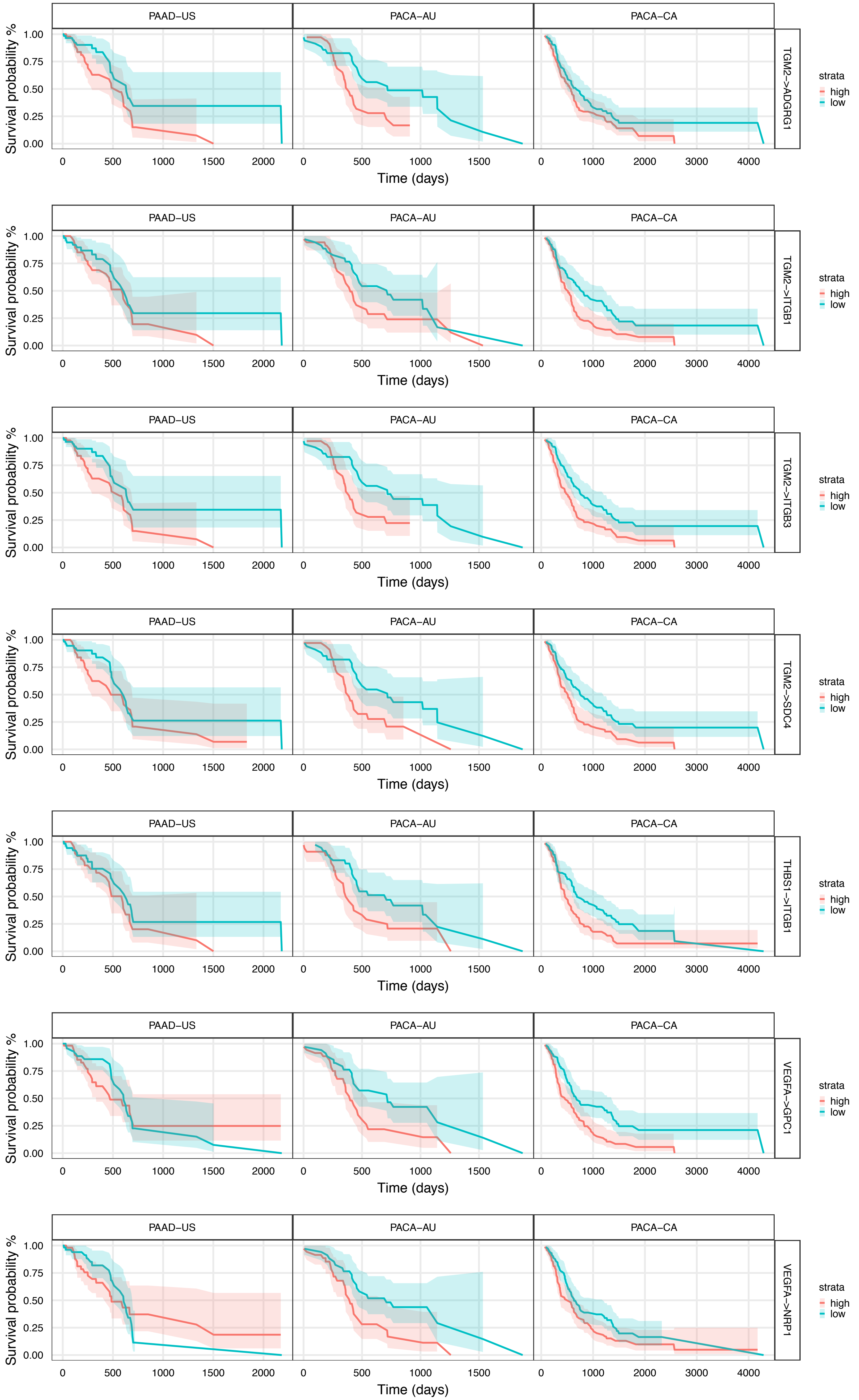
PAAD–US
PACA–AU
PACA–CA
Survival probability %
Time (days)
strata
high
low
TGM2–>ADGRG1
TGM2–>ITGB1
TGM2–>ITGB3
TGM2–>SDC4
THBS1–>ITGB1
VEGFA–>GPC1
VEGFA–>NRP1
0.00
0.25
0.50
0.75
1.00
0
500
1000
1500
2000
3000
4000

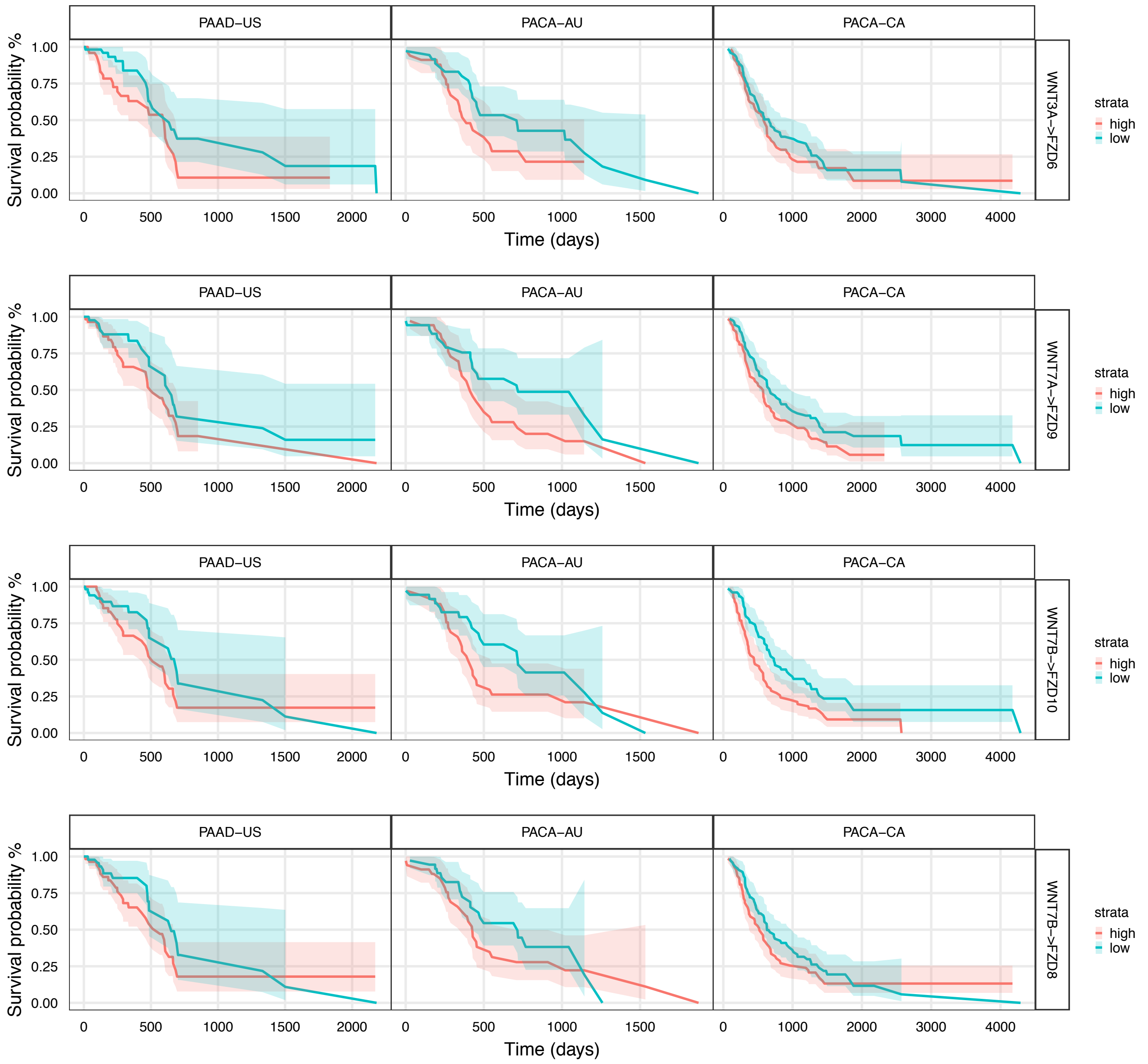

Figure S1. Kaplan-Meier plots of poor-prognostic LR pairs.

Kaplan-Meier plots of poor-prognostic LR pairs for each cohort. Hazard ratios, p-values, and q-values are shown in **Table S2**.

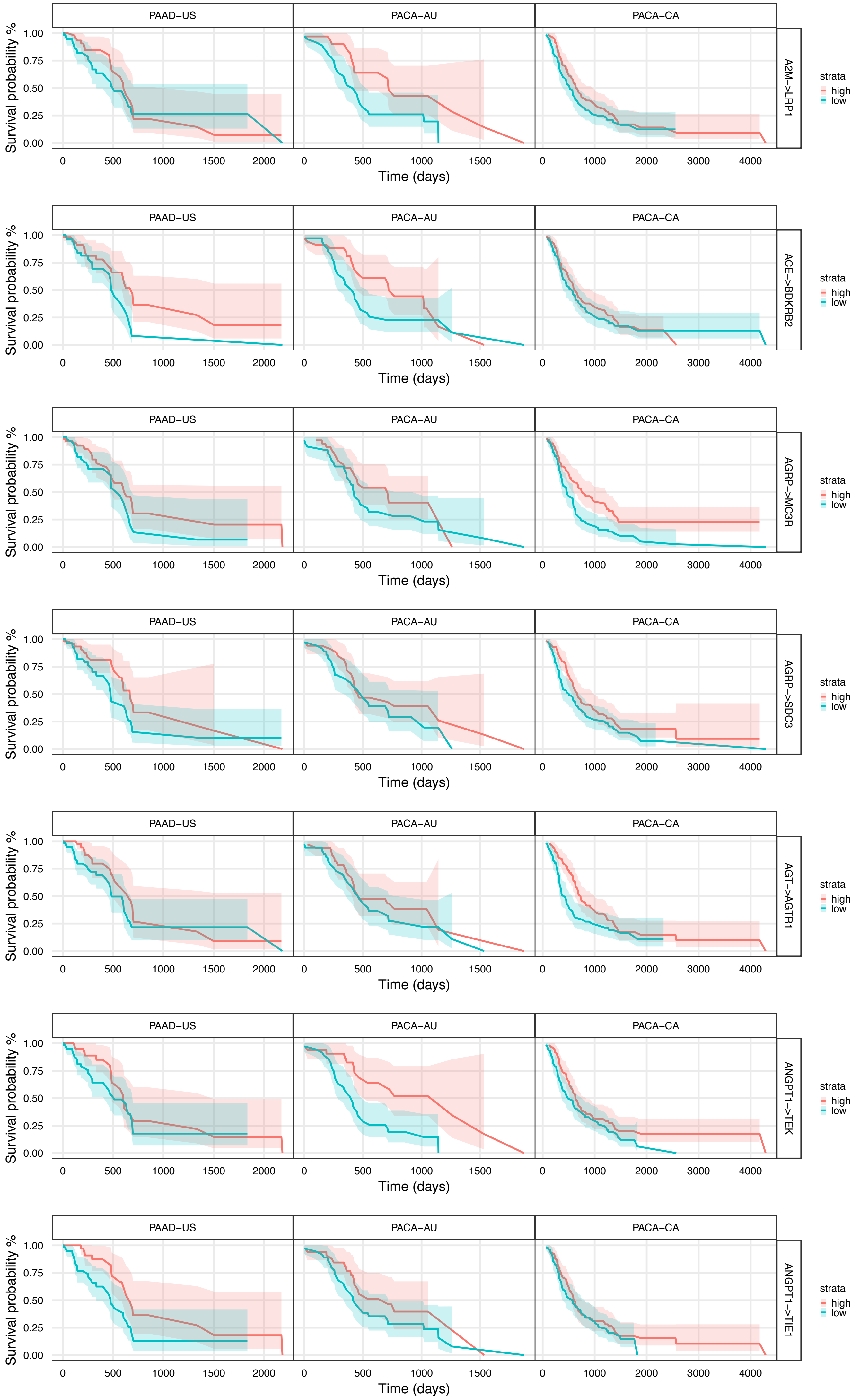
PAAD–US
PACA–AU
PACA–CA
Survival probability %
Time (days)
strata
high
low
A2M–>LRP1
ACE–>BDKRB2
AGRP–>MC3R
AGRP–>SDC3
AGT–>AGTR1
ANGPT1–>TEK
ANGPT1–>TIE1

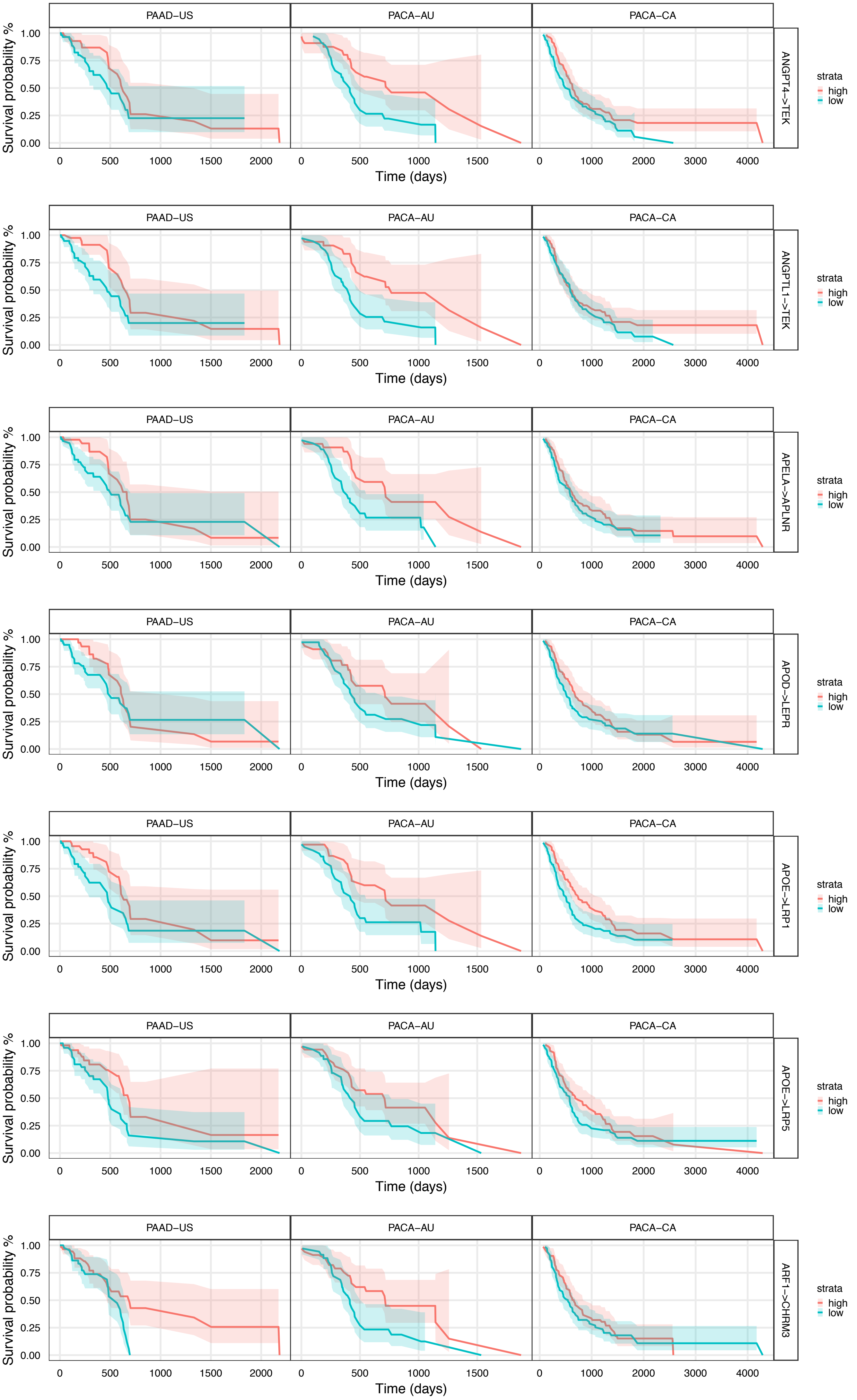
PAAD–US
PACA–AU
PACA–CA
ANGPT4–>TEK
ANGPTL1–>TEK
APELA–>APLNR
APOD–>LEPR
APOE–>LRP1
APOE–>LRP5
ARF1–>CHRM3
Survival probability %
1.00
0.75
0.50
0.25
0.00
0
500
1000
1500
2000
2000
3000
4000
Time (days)
strata
high
low

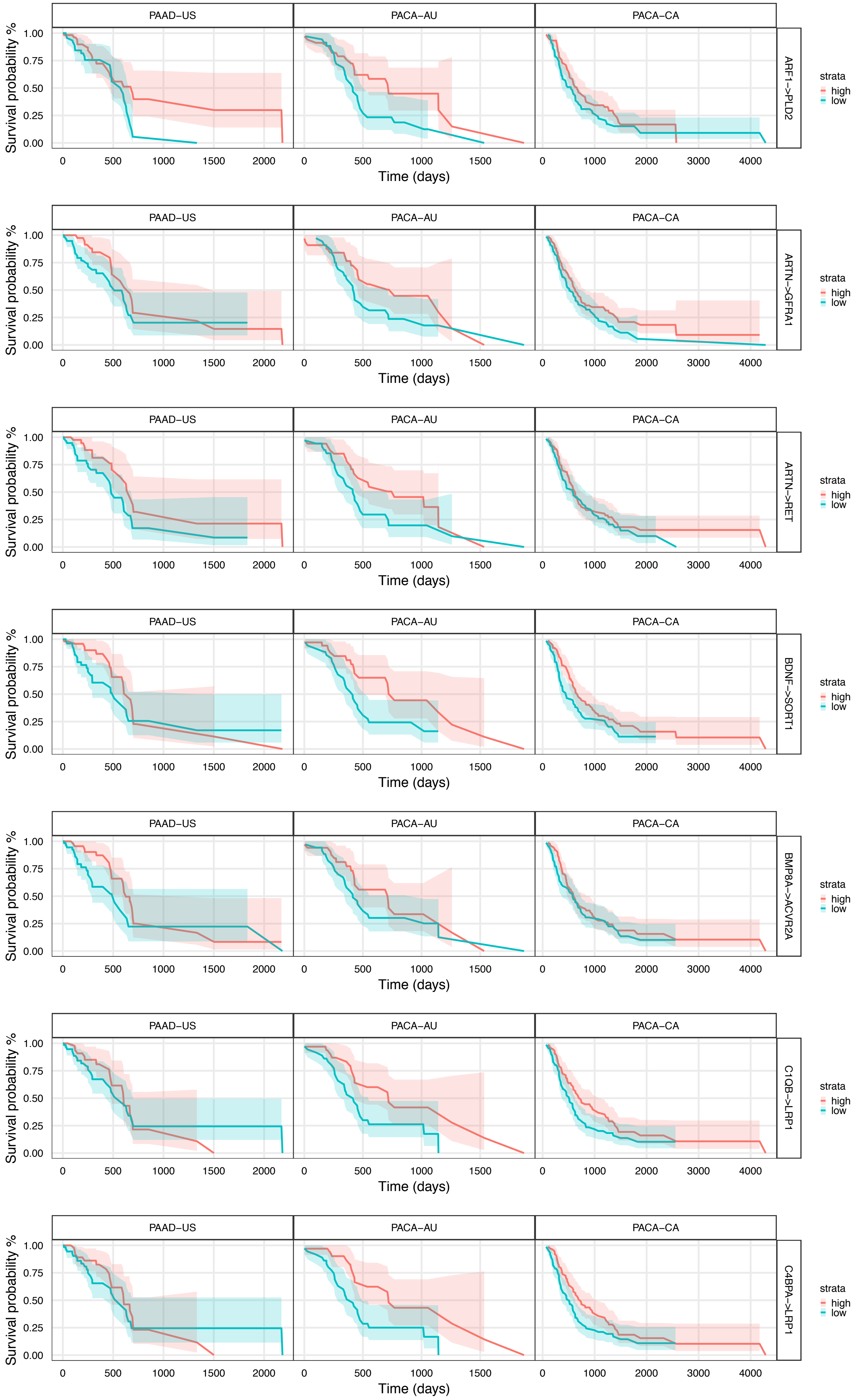
PAAD−US
PACA−AU
PACA−CA
Survival probability %
1.00
0.75
0.50
0.25
0.00
0
500
1000
1500
2000
0
500
1000
1500
0
1000
2000
3000
4000
Time (days)
ARF1−>PLD2
ARTN−>GFRA1
ARTN−>RET
BDNF−>SORT1
BMP8A−>ACVR2A
C1QB−>LRP1
C4BPA−>LRP1
strata
high
low

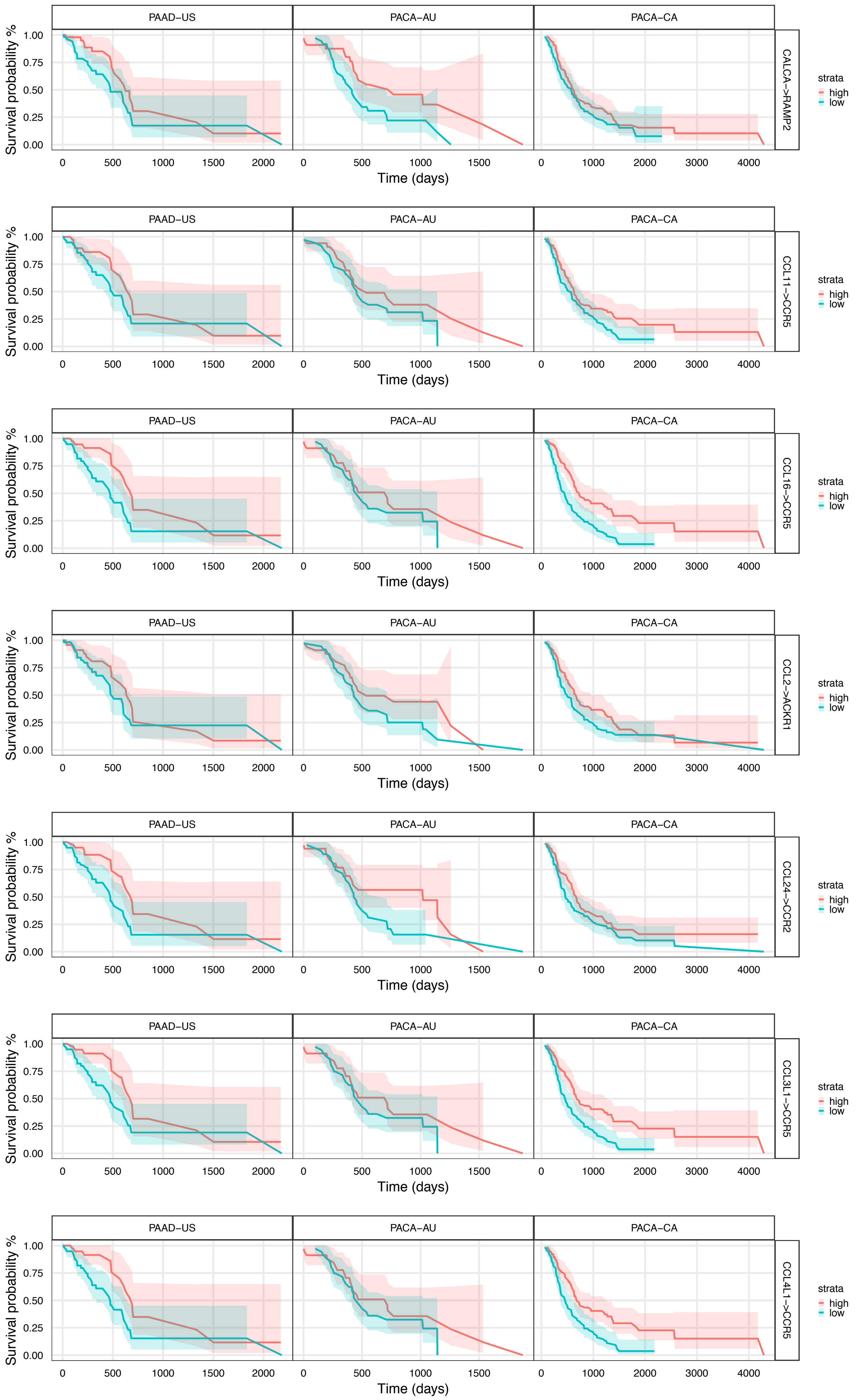
PAAD–US
PACA–AU
PACA–CA
Survival probability %
1.00
0.75
0.50
0.25
0.00
0
500
1000
1500
2000
2000
3000
4000
Time (days)
strata
high
low
CALCA–>RAMP2
CCL11–>CCR5
CCL16–>CCR5
CCL2–>ACKR1
CCL24–>CCR2
CCL3L1–>CCR5
CCL4L1–>CCR5

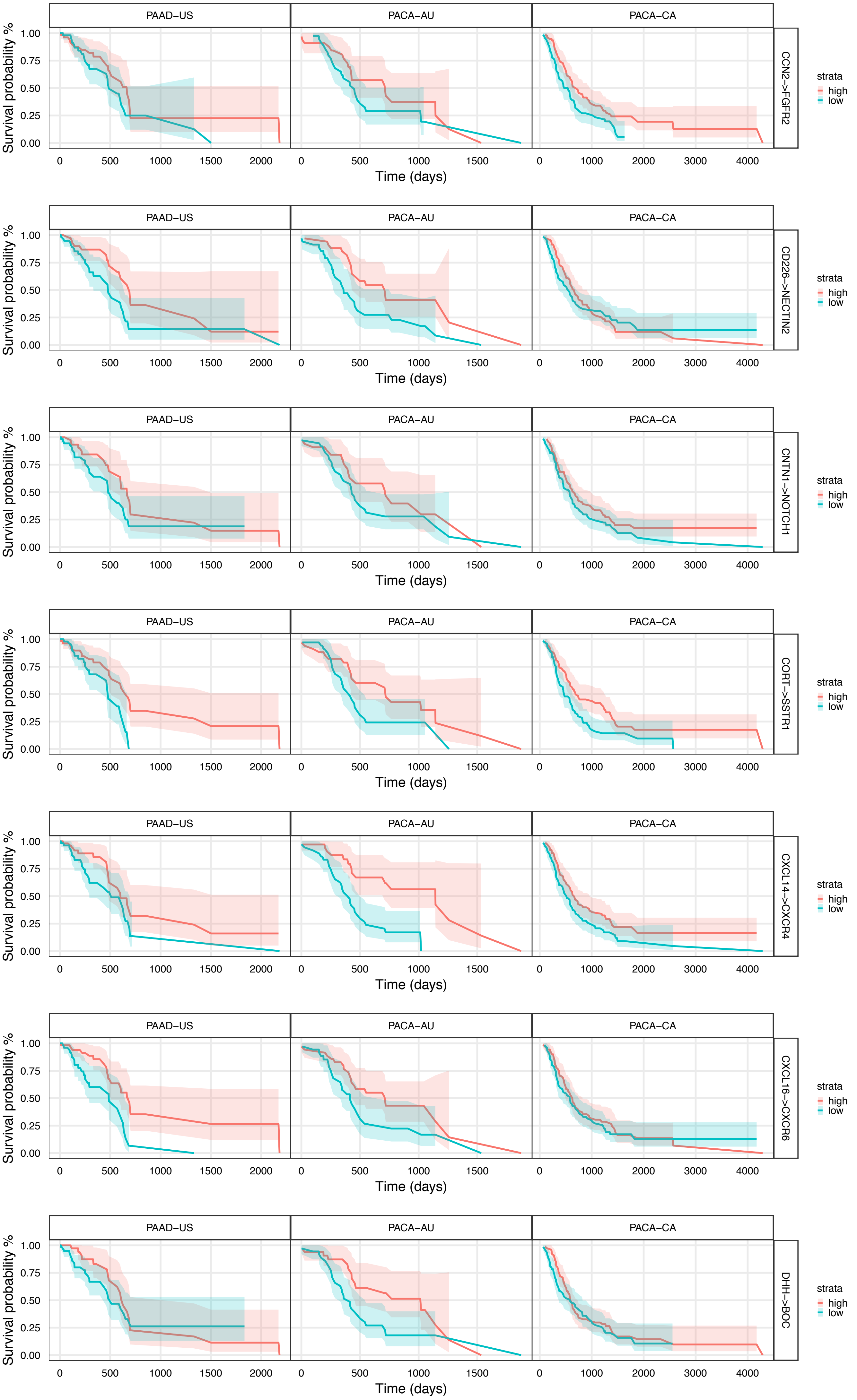
PAAD−US
PACA−AU
PACA−CA
Survival probability %
Time (days)
strata
high
low
CCN2−>FGFR2
CD226−>NECTIN2
CNTN1−>NOTCH1
CORT−>SSTR1
CXCL14−>CXCR4
CXCL16−>CXCR6
DHH−>BOC

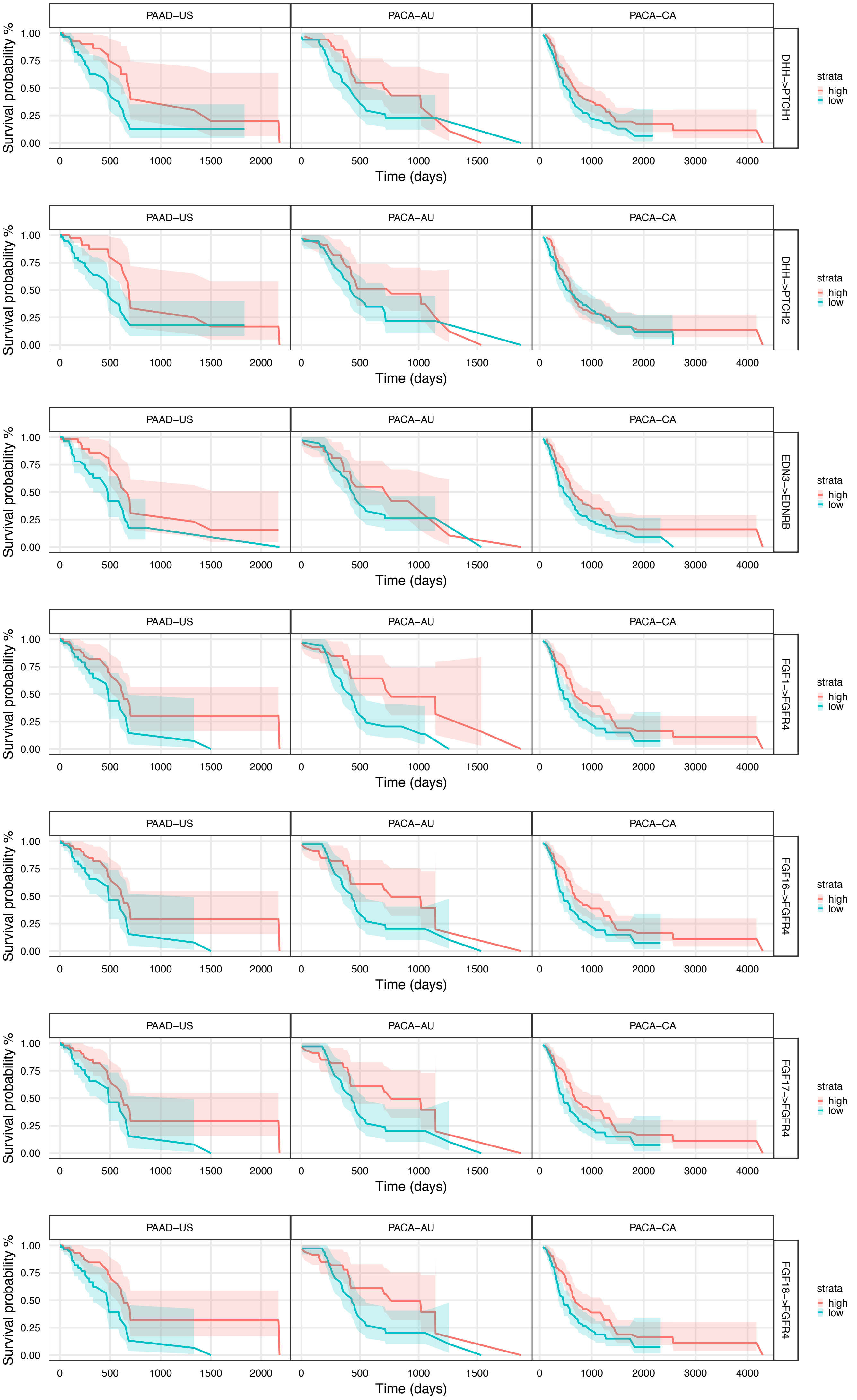
PAAD–US
PACA–AU
PACA–CA
DHH–>PTCH1
DHH–>PTCH2
EDN3–>EDNRB
FGF1–>FGFR4
FGF16–>FGFR4
FGF17–>FGFR4
FGF18–>FGFR4
Survival probability %
1.00
0.75
0.50
0.25
0.00
0
500
1000
1500
2000
3000
4000
Time (days)
strata
high
low

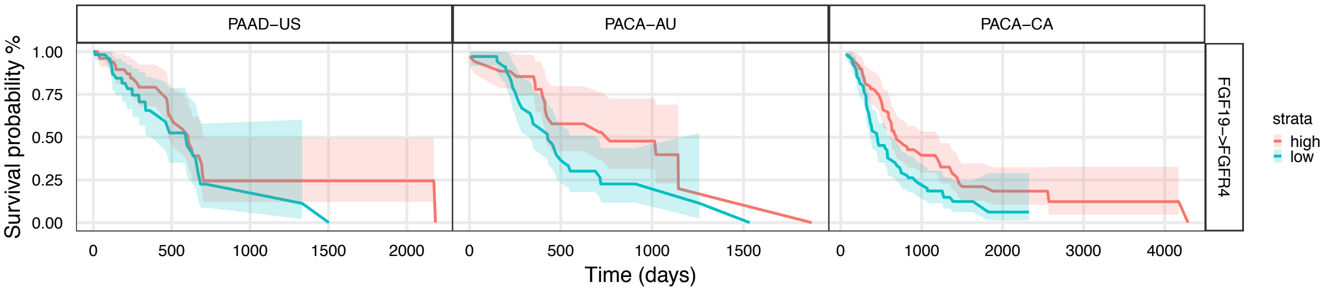
PAAD–US
PACA–AU
PACA–CA
FGF19–>FGFR4
Survival probability %
1.00
0.75
0.50
0.25
0.00
0
500
1000
1500
2000
3000
4000
Time (days)
strata
high
low

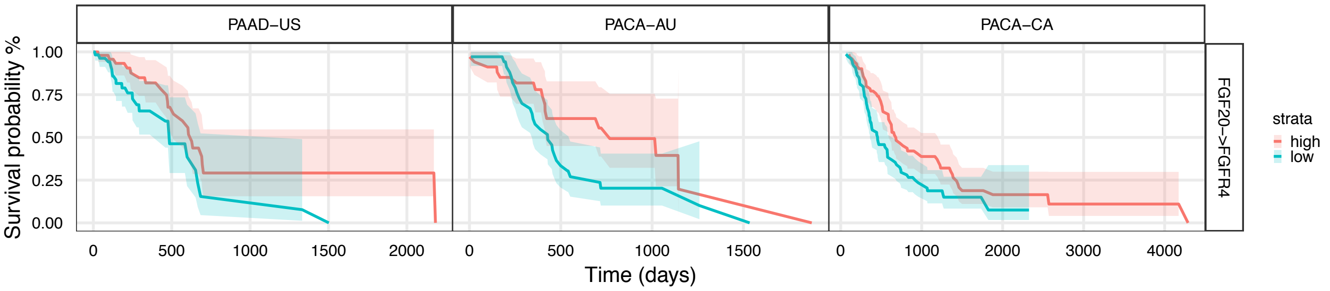
PAAD–US
PACA–AU
PACA–CA
FGF20–>FGFR4
Survival probability %
1.00
0.75
0.50
0.25
0.00
0
500
1000
1500
2000
3000
4000
Time (days)
strata
high
low

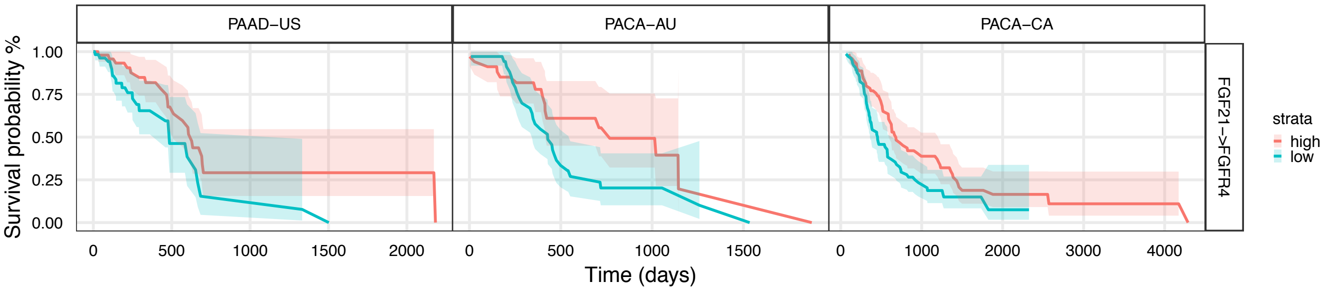
PAAD–US
PACA–AU
PACA–CA
FGF21–>FGFR4
Survival probability %
1.00
0.75
0.50
0.25
0.00
0
500
1000
1500
2000
3000
4000
Time (days)
strata
high
low

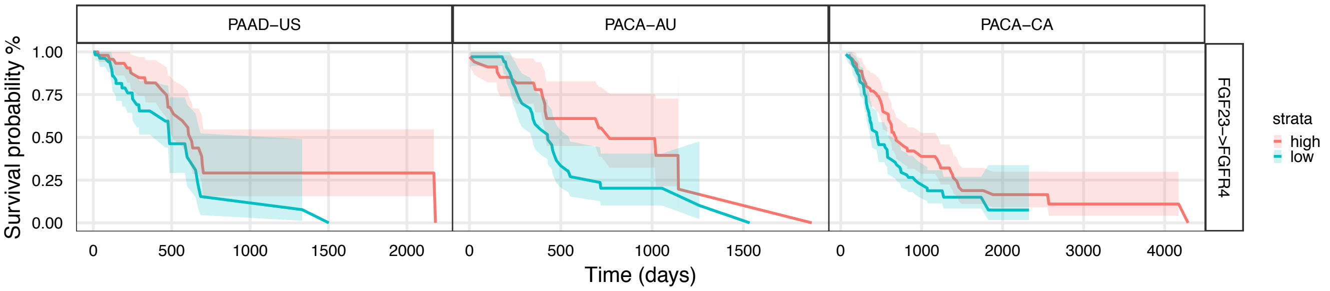
PAAD–US
PACA–AU
PACA–CA
FGF23–>FGFR4
Survival probability %
1.00
0.75
0.50
0.25
0.00
0
500
1000
1500
2000
3000
4000
Time (days)
strata
high
low

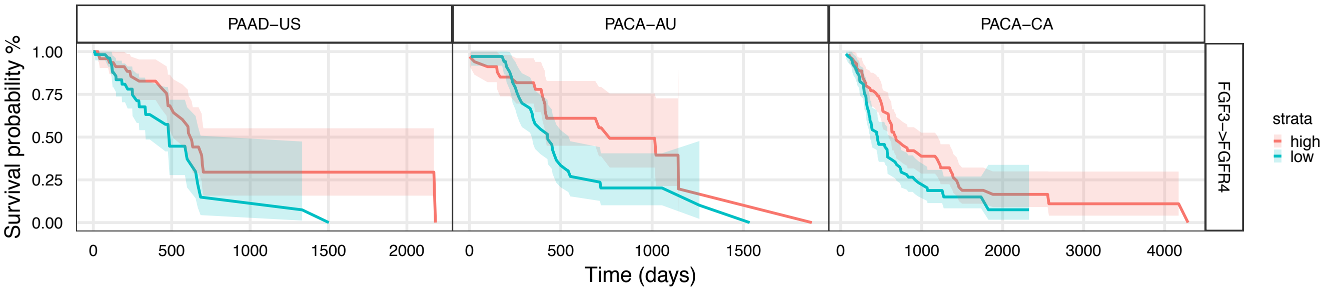
PAAD–US
PACA–AU
PACA–CA
FGF3–>FGFR4
Survival probability %
1.00
0.75
0.50
0.25
0.00
0
500
1000
1500
2000
3000
4000
Time (days)
strata
high
low

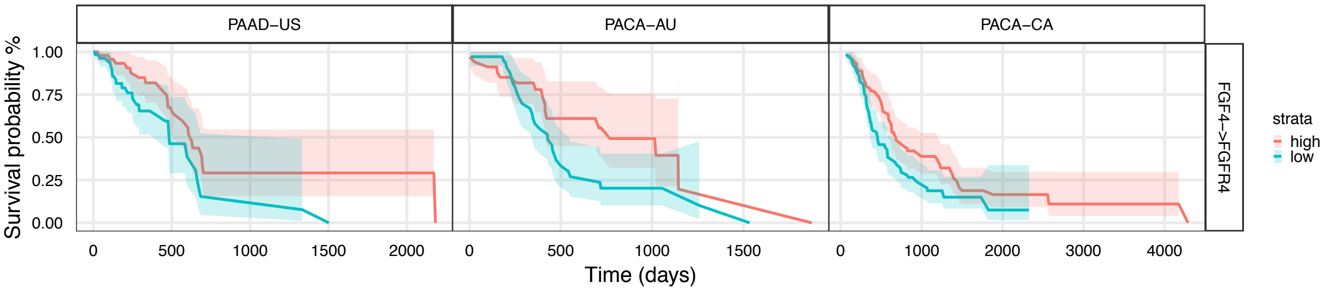
PAAD–US
PACA–AU
PACA–CA
FGF4–>FGFR4
Survival probability %
1.00
0.75
0.50
0.25
0.00
0
500
1000
1500
2000
3000
4000
Time (days)
strata
high
low

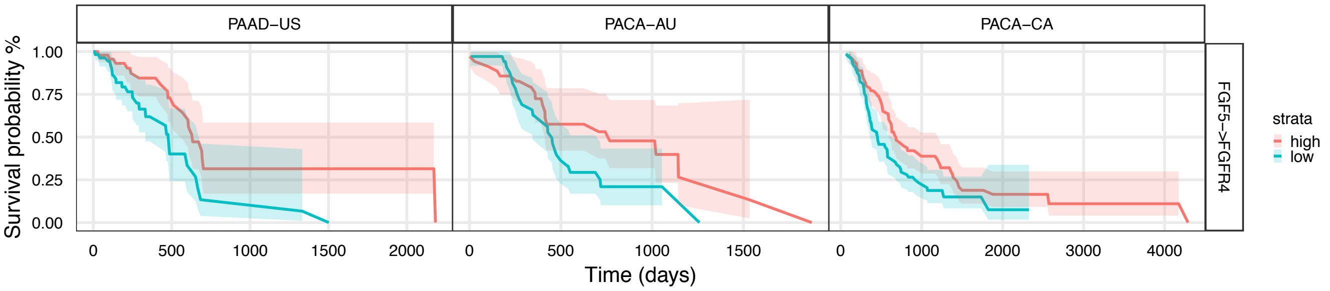
PAAD–US
PACA–AU
PACA–CA
FGF5–>FGFR4
Survival probability %
1.00
0.75
0.50
0.25
0.00
0
500
1000
1500
2000
3000
4000
Time (days)
strata
high
low

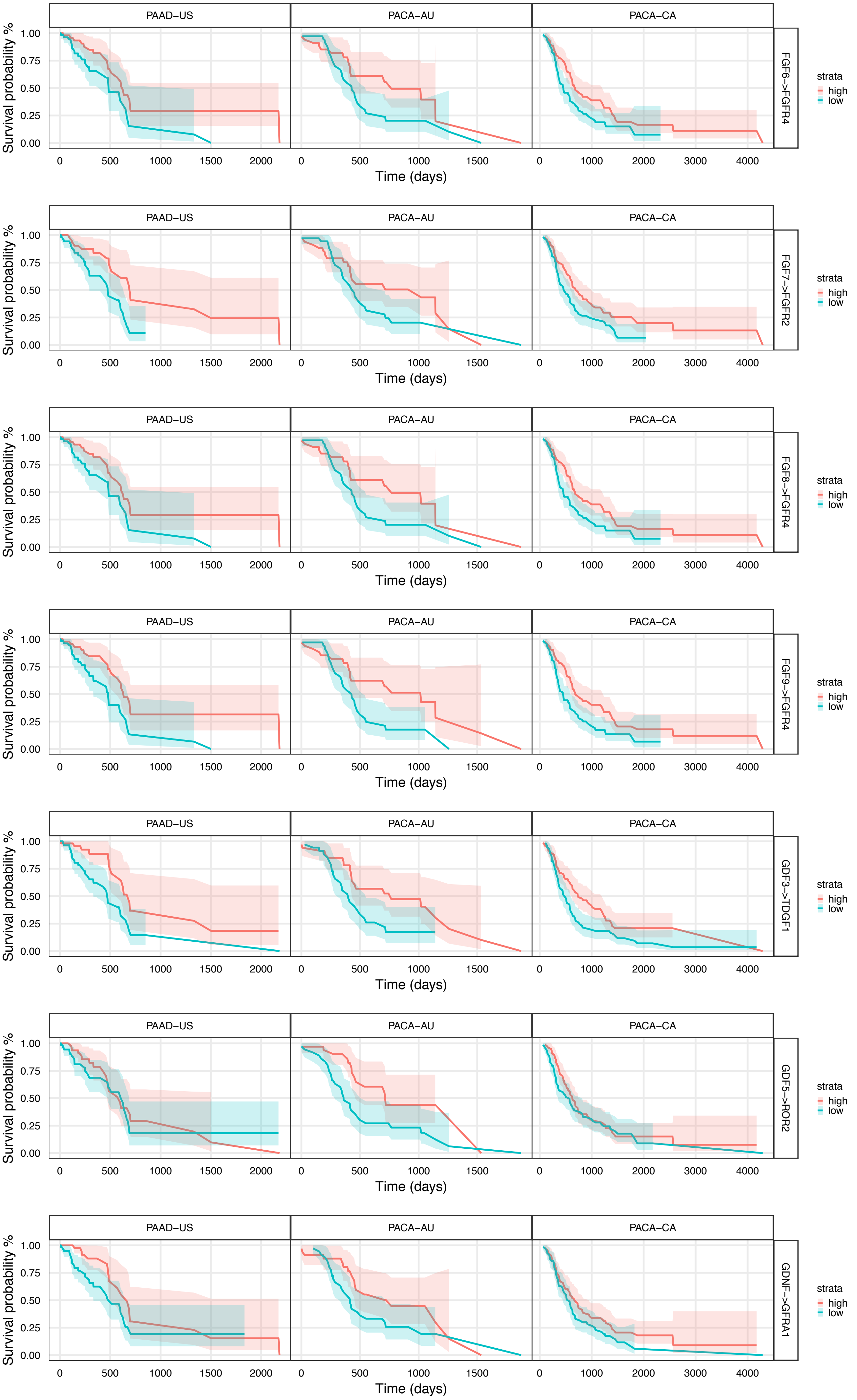
PAAD−US
PACA−AU
PACA−CA
Survival probability %
Time (days)
strata
high
low
FGF6−>FGFR4
FGF7−>FGFR2
FGF8−>FGFR4
FGF9−>FGFR4
GDF3−>TDGF1
GDF5−>ROR2
GDNF−>GFRA1

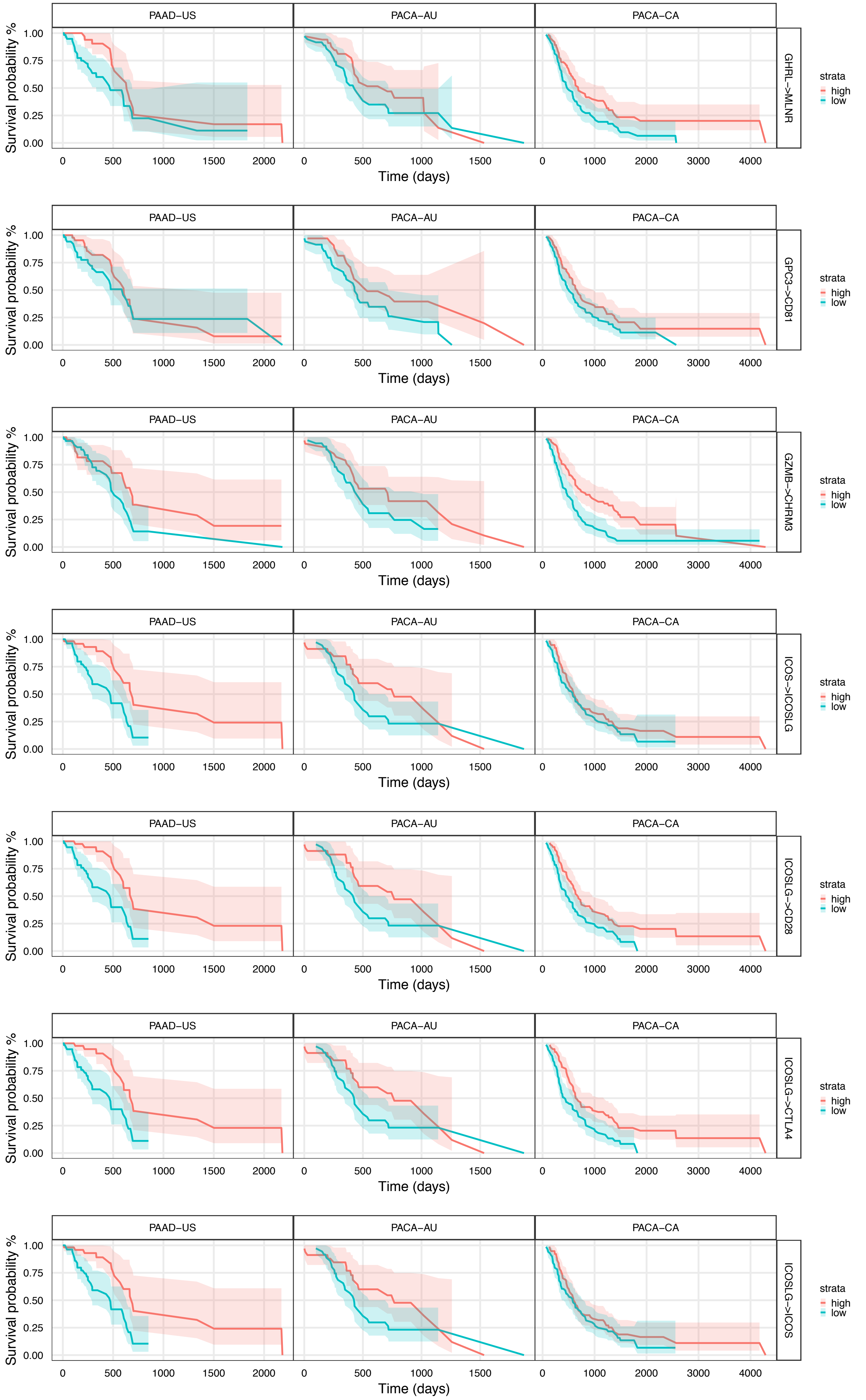
PAAD−US
PACA−AU
PACA−CA
Survival probability %
1.00
0.75
0.50
0.25
0.00
0
500
1000
1500
2000
3000
4000
Time (days)
strata
high
low
GHRL−>MLNR
GPC3−>CD81
GZMB−>CHRM3
ICOS−>ICOSLG
ICOSLG−>CD28
ICOSLG−>CTLA4
ICOSLG−>ICOS

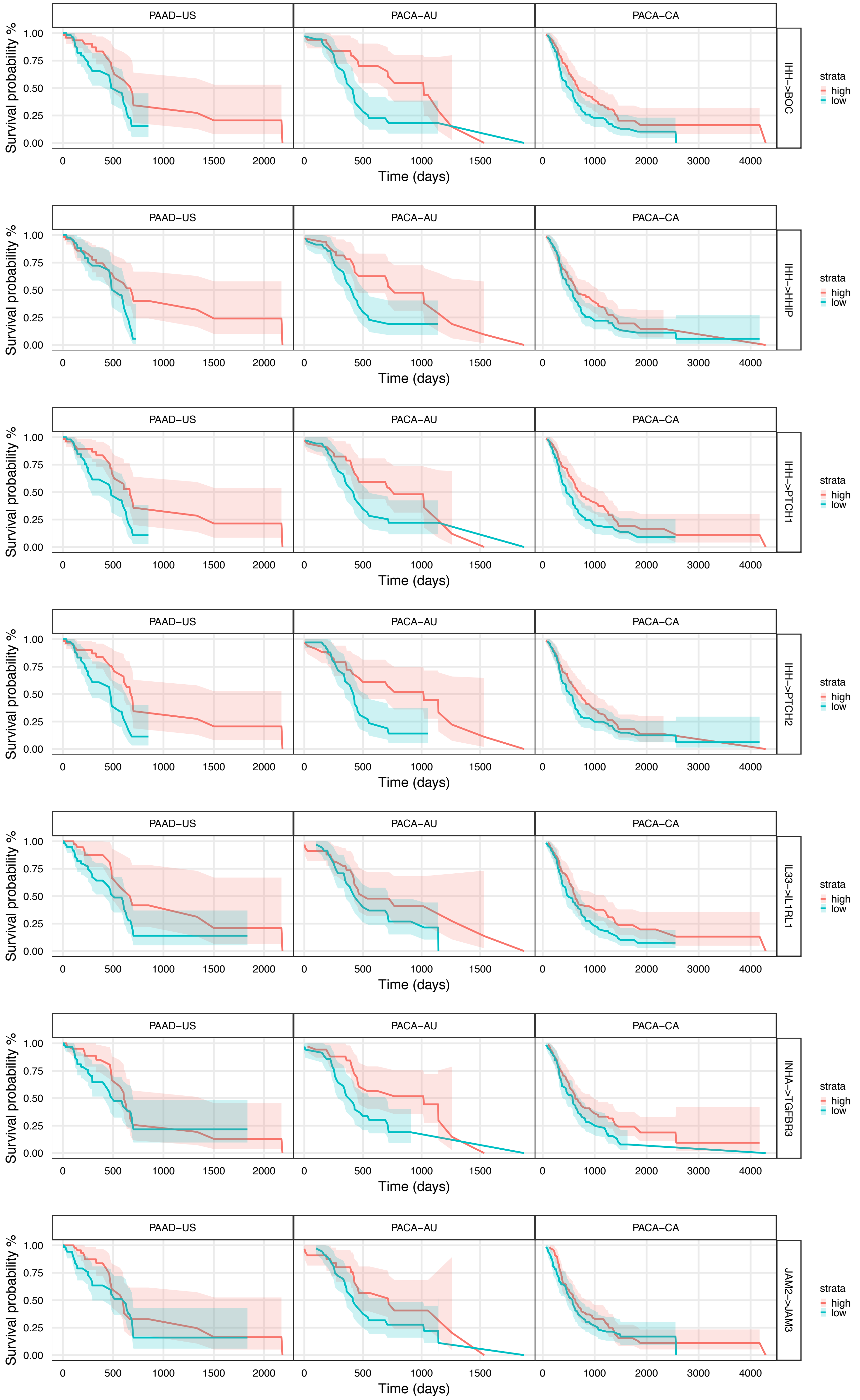
PAAD–US
PACA–AU
PACA–CA
Survival probability %
Time (days)
strata
high
low
IHH–>BOC
IHH–>HHIP
IHH–>PTCH1
IHH–>PTCH2
IL33–>IL1RL1
INHA–>TGFBR3
JAM2–>JAM3

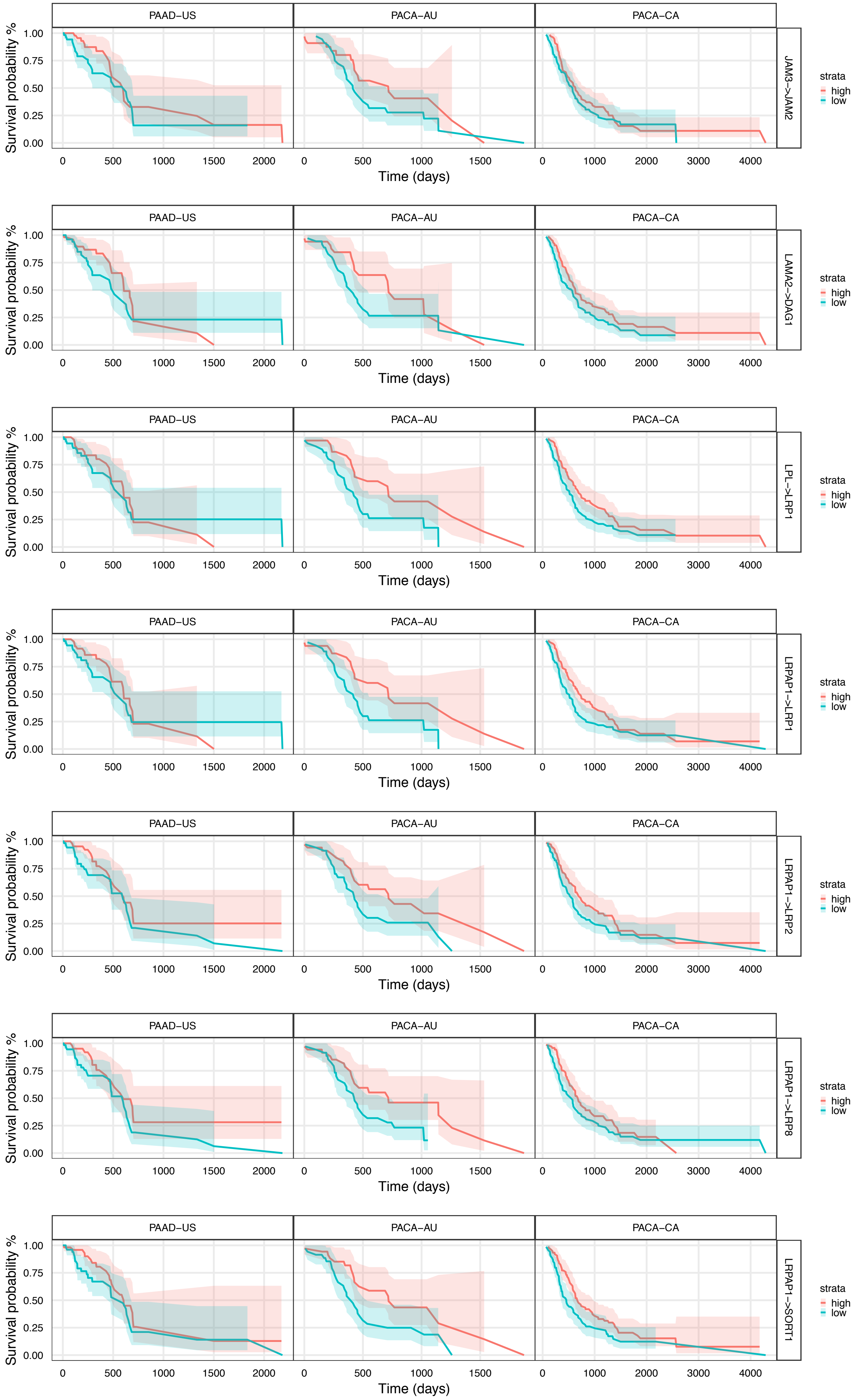
PAAD−US
PACA−AU
PACA−CA
Survival probability %
Time (days)
strata
high
low
JAM3−>JAM2
LAMA2−>DAG1
LPL−>LRP1
LRPAP1−>LRP1
LRPAP1−>LRP2
LRPAP1−>LRP8
LRPAP1−>SORT1
0.00
0.25
0.50
0.75
1.00
0
500
1000
1500
2000
3000
4000

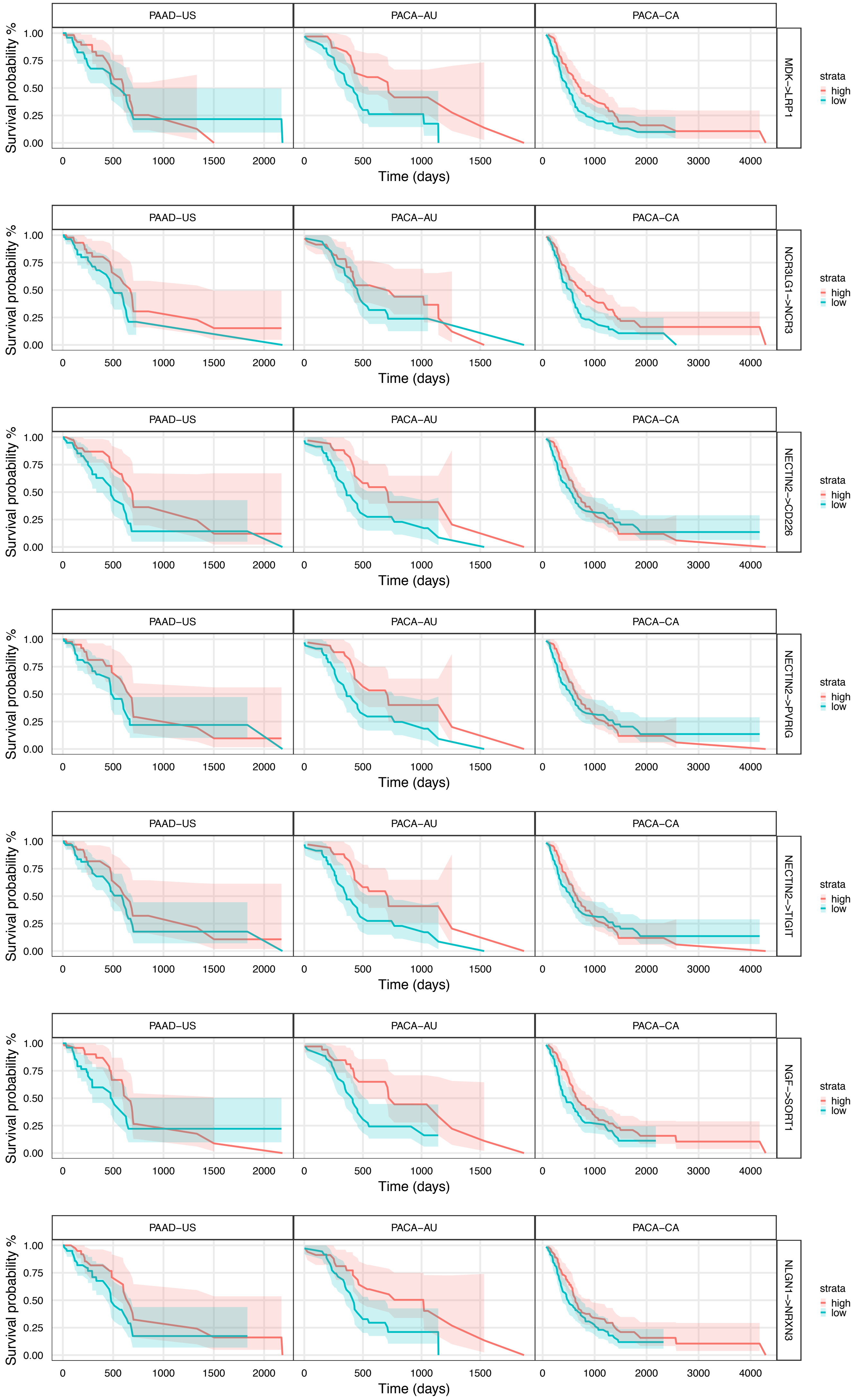
PAAD–US
PACA–AU
PACA–CA
MDK–>LRP1
NCR3LG1–>NCR3
NECTIN2–>CD226
NECTIN2–>PVRIG
NECTIN2–>TIGIT
NGF–>SORT1
NLGN1–>NRXN3
Survival probability %
Time (days)
strata
high
low
1.00
0.75
0.50
0.25
0.00
0
500
1000
1500
2000
3000
4000

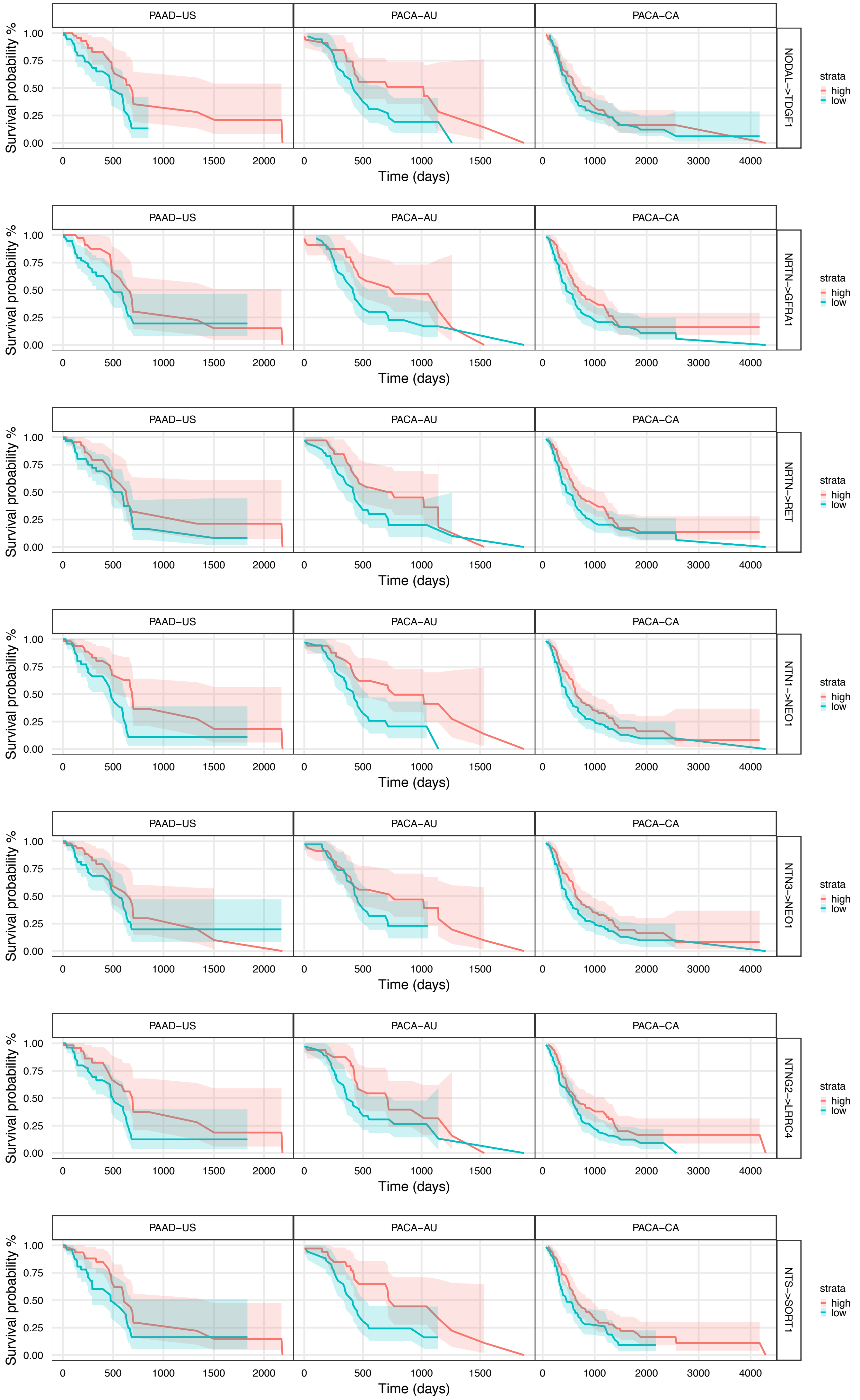
PAAD–US
PACA–AU
PACA–CA
NODAL–>TDGF1
NRTN–>GFRA1
NRTN–>RET
NTN1–>NEO1
NTN3–>NEO1
NTNG2–>LRRC4
NTS–>SORT1
Survival probability %
1.00
0.75
0.50
0.25
0.00
0 500 1000 1500 2000
0 500 1000 1500
0 1000 2000 3000 4000
Time (days)
strata
high
low

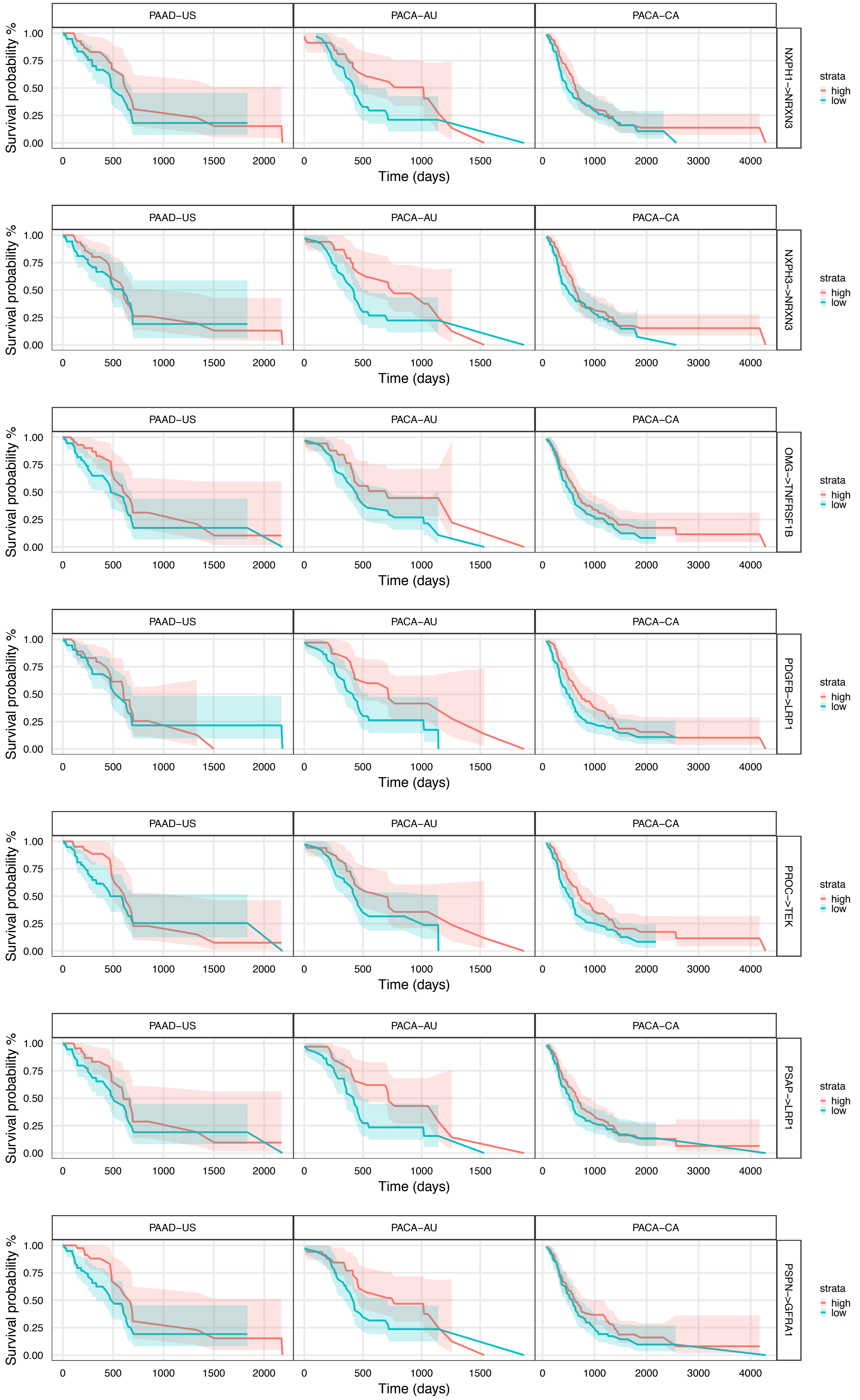
PAAD–US
PACA–AU
PACA–CA
Survival probability %
1.00
0.75
0.50
0.25
0.00
Time (days)
0
500
1000
1500
2000
3000
4000
strata
high
low
NXPH1–>NRXN3
NXPH3–>NRXN3
OMG–>TNFRSF1B
PDGFB–>LRP1
PROC–>TEK
PSAP–>LRP1
PSPN–>GFRA1

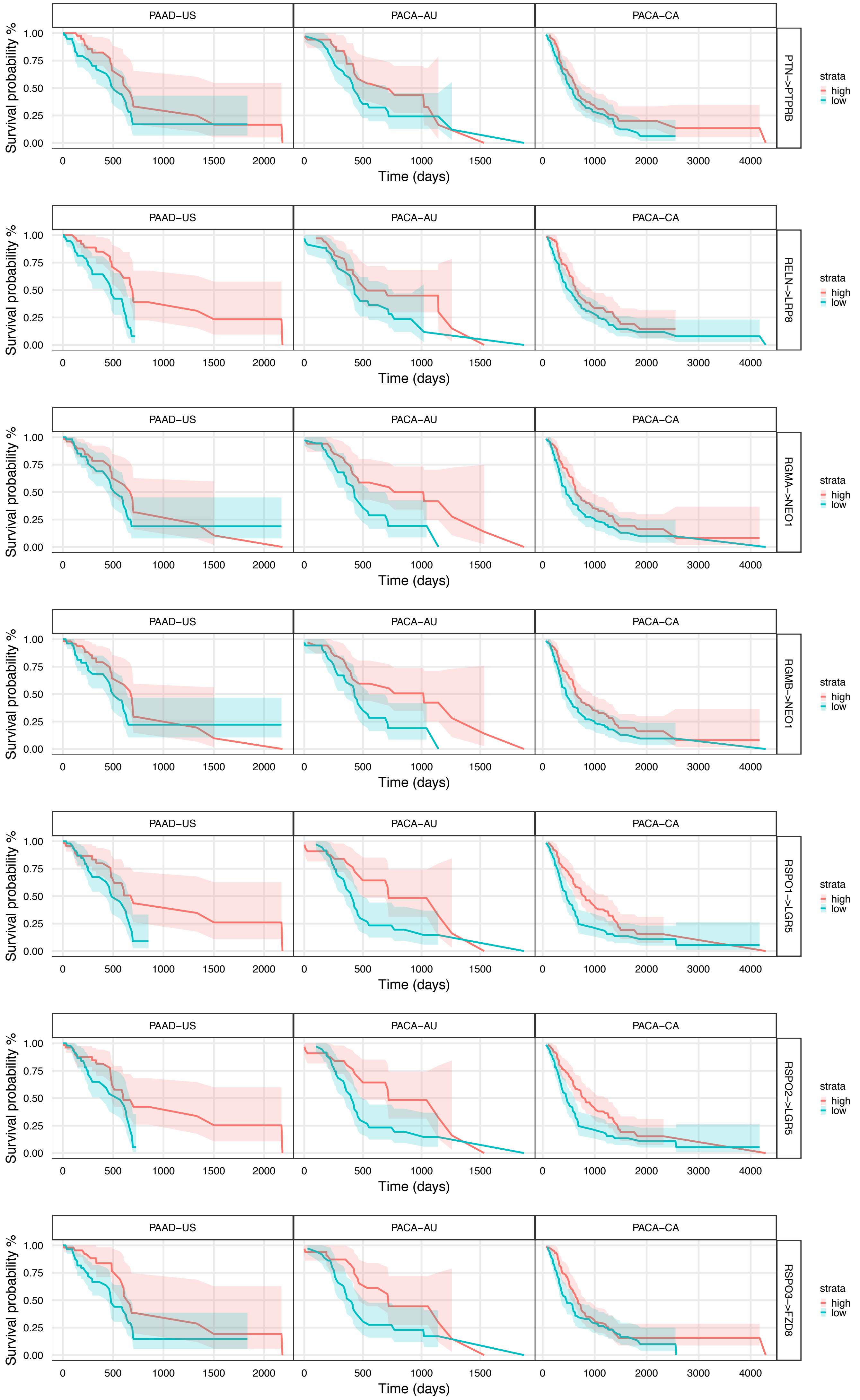
PAAD−US
PACA−AU
PACA−CA
Survival probability %
Time (days)
strata
high
low
PTN−>PTPRB
RELN−>LRP8
RGMA−>NEO1
RGMB−>NEO1
RSPO1−>LGR5
RSPO2−>LGR5
RSPO3−>FZD8

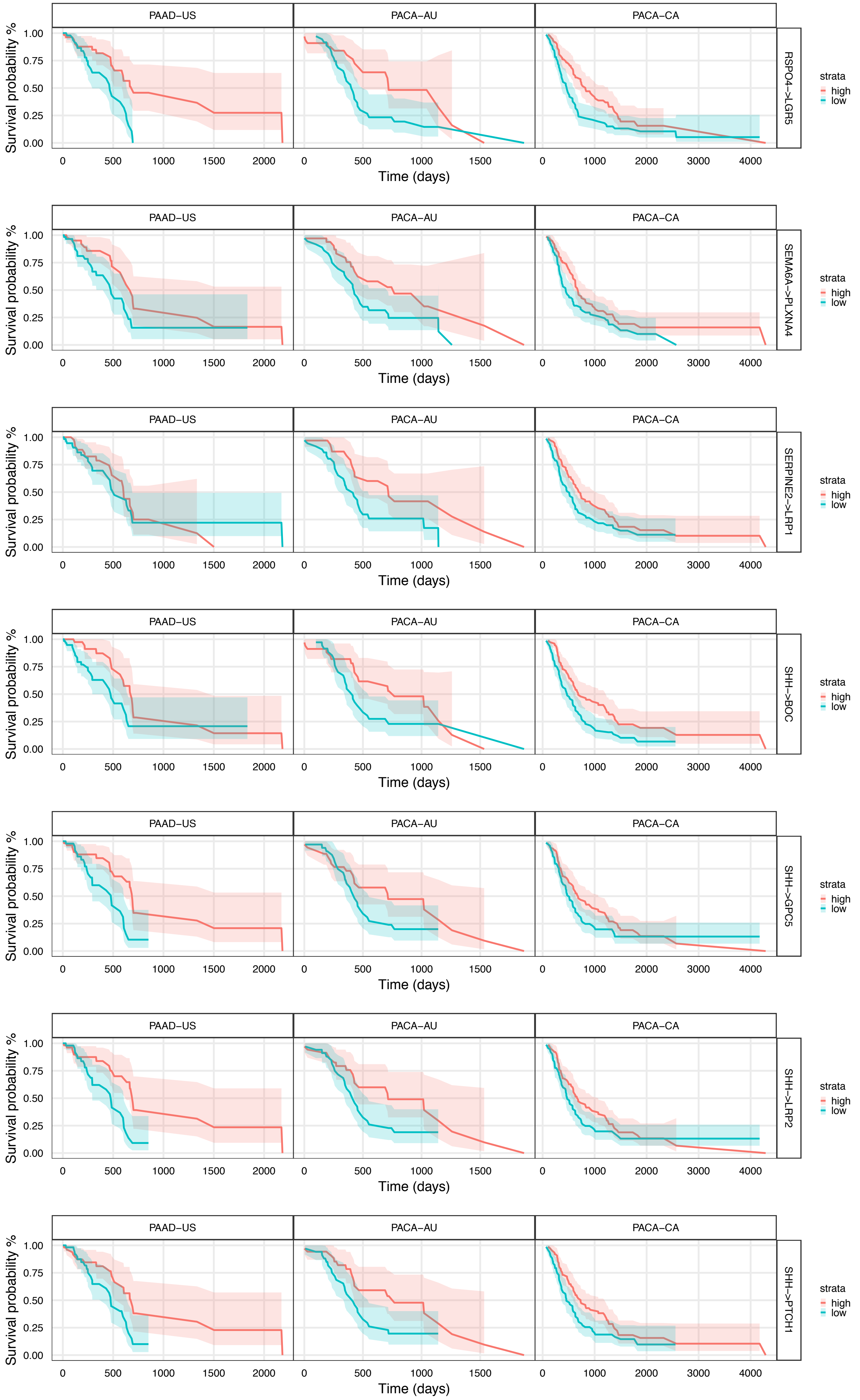
PAAD–US
PACA–AU
PACA–CA
RSPO4–>LGR5
SEMA6A–>PLXNA4
SERPINE2–>LRP1
SHH–>BOC
SHH–>GPC5
SHH–>LRP2
SHH–>PTCH1
Survival probability %
Time (days)
strata
high
low
0.00
0.25
0.50
0.75
1.00
0
500
1000
1500
2000
3000
4000

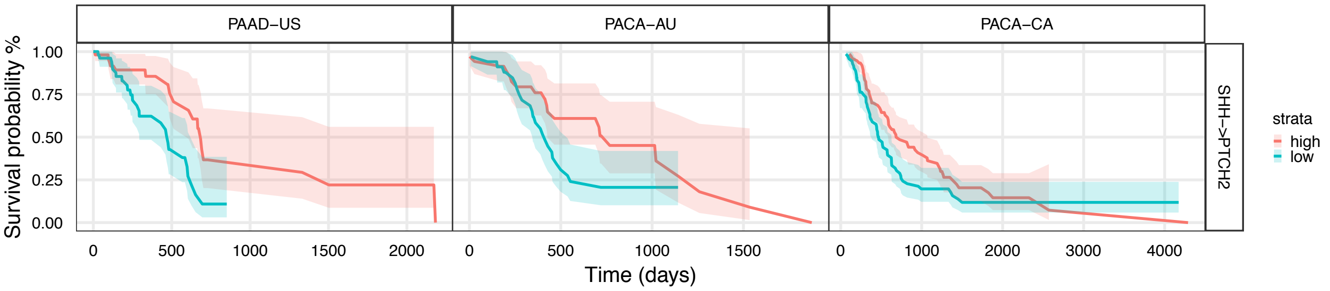
PAAD–US
PACA–AU
PACA–CA
Survival probability %
1.00
0.75
0.50
0.25
0.00
0 500 1000 1500 2000
0 500 1000 1500
0 1000 2000 3000 4000
Time (days)
SHH–>PTCH2
strata
high
low

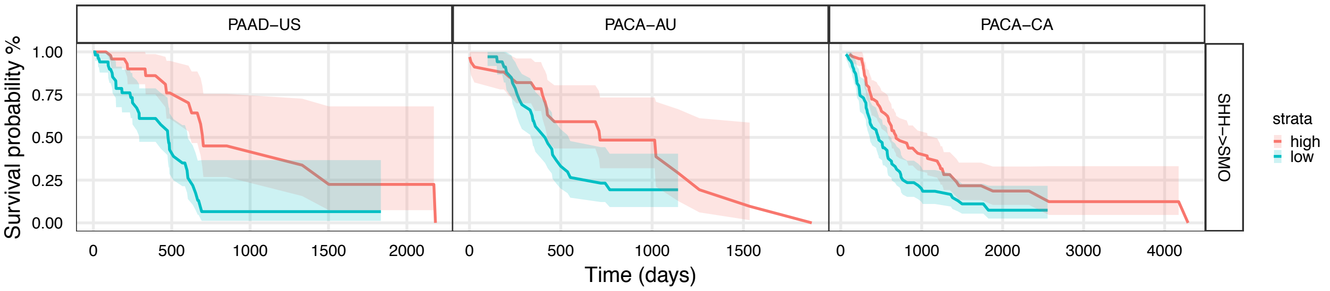
PAAD–US
PACA–AU
PACA–CA
Survival probability %
1.00
0.75
0.50
0.25
0.00
0 500 1000 1500 2000
0 500 1000 1500
0 1000 2000 3000 4000
Time (days)
SHH–>SMO
strata
high
low

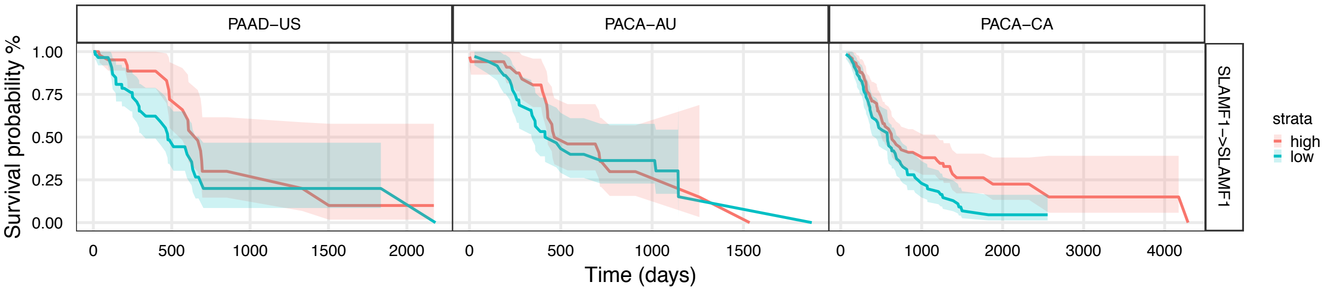
PAAD–US
PACA–AU
PACA–CA
Survival probability %
1.00
0.75
0.50
0.25
0.00
0 500 1000 1500 2000
0 500 1000 1500
0 1000 2000 3000 4000
Time (days)
SLAMF1–>SLAMF1
strata
high
low

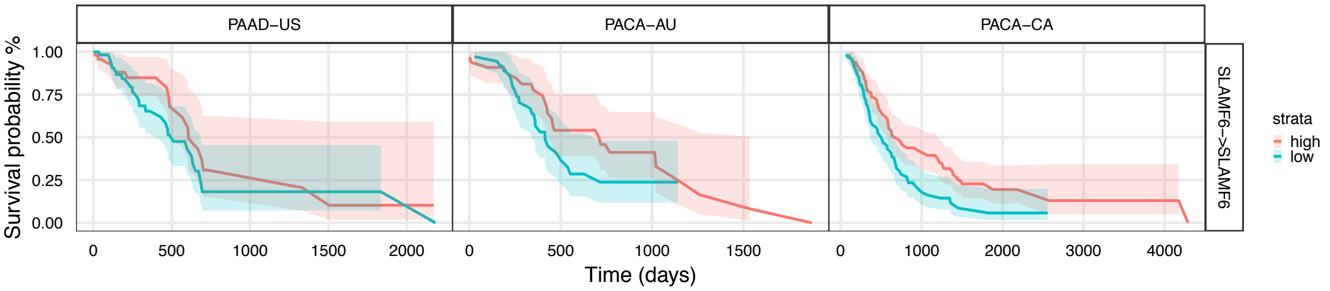
PAAD–US
PACA–AU
PACA–CA
Survival probability %
1.00
0.75
0.50
0.25
0.00
0 500 1000 1500 2000
0 500 1000 1500
0 1000 2000 3000 4000
Time (days)
SLAMF6–>SLAMF6
strata
high
low

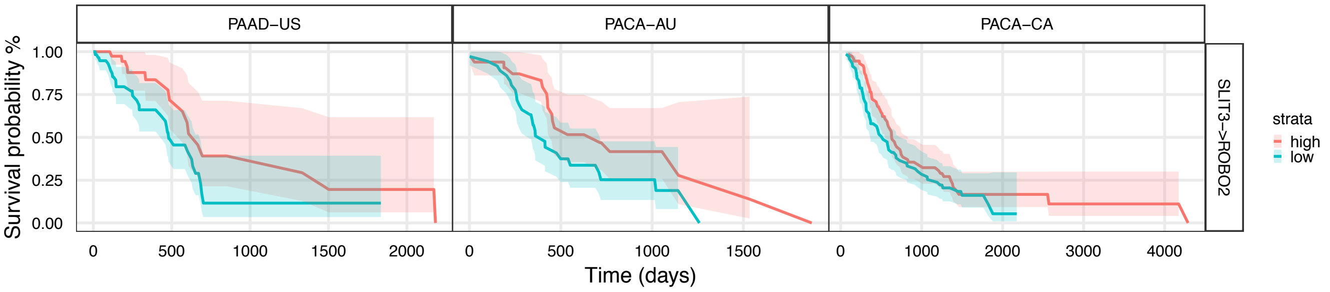
PAAD–US
PACA–AU
PACA–CA
Survival probability %
1.00
0.75
0.50
0.25
0.00
0 500 1000 1500 2000
0 500 1000 1500
0 1000 2000 3000 4000
Time (days)
SLIT3–>ROBO2
strata
high
low

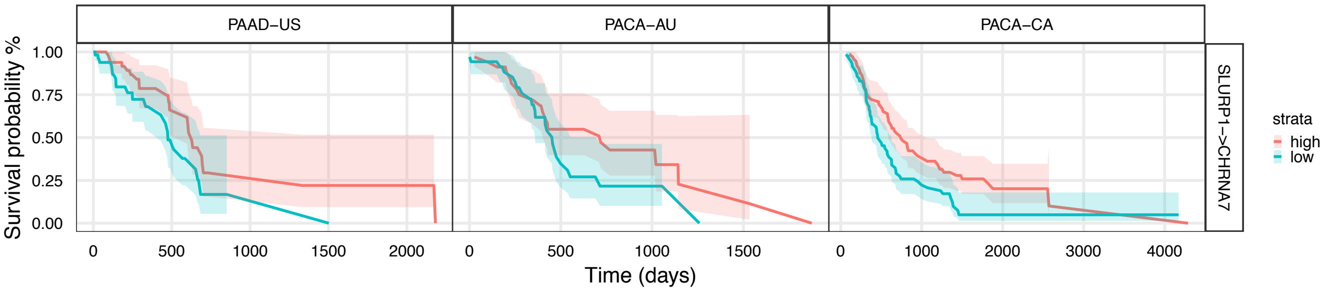
PAAD–US
PACA–AU
PACA–CA
Survival probability %
1.00
0.75
0.50
0.25
0.00
0 500 1000 1500 2000
0 500 1000 1500
0 1000 2000 3000 4000
Time (days)
SLURP1–>CHRNA7
strata
high
low

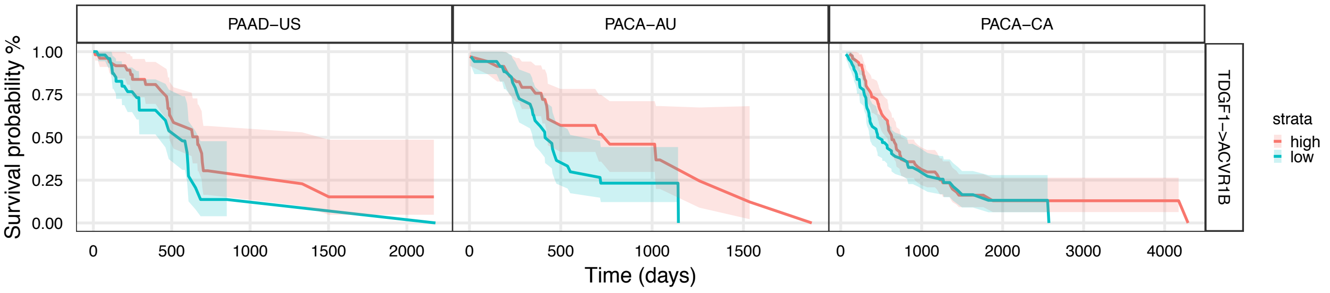
PAAD–US
PACA–AU
PACA–CA
Survival probability %
1.00
0.75
0.50
0.25
0.00
0 500 1000 1500 2000
0 500 1000 1500
0 1000 2000 3000 4000
Time (days)
TDGF1–>ACVR1B
strata
high
low

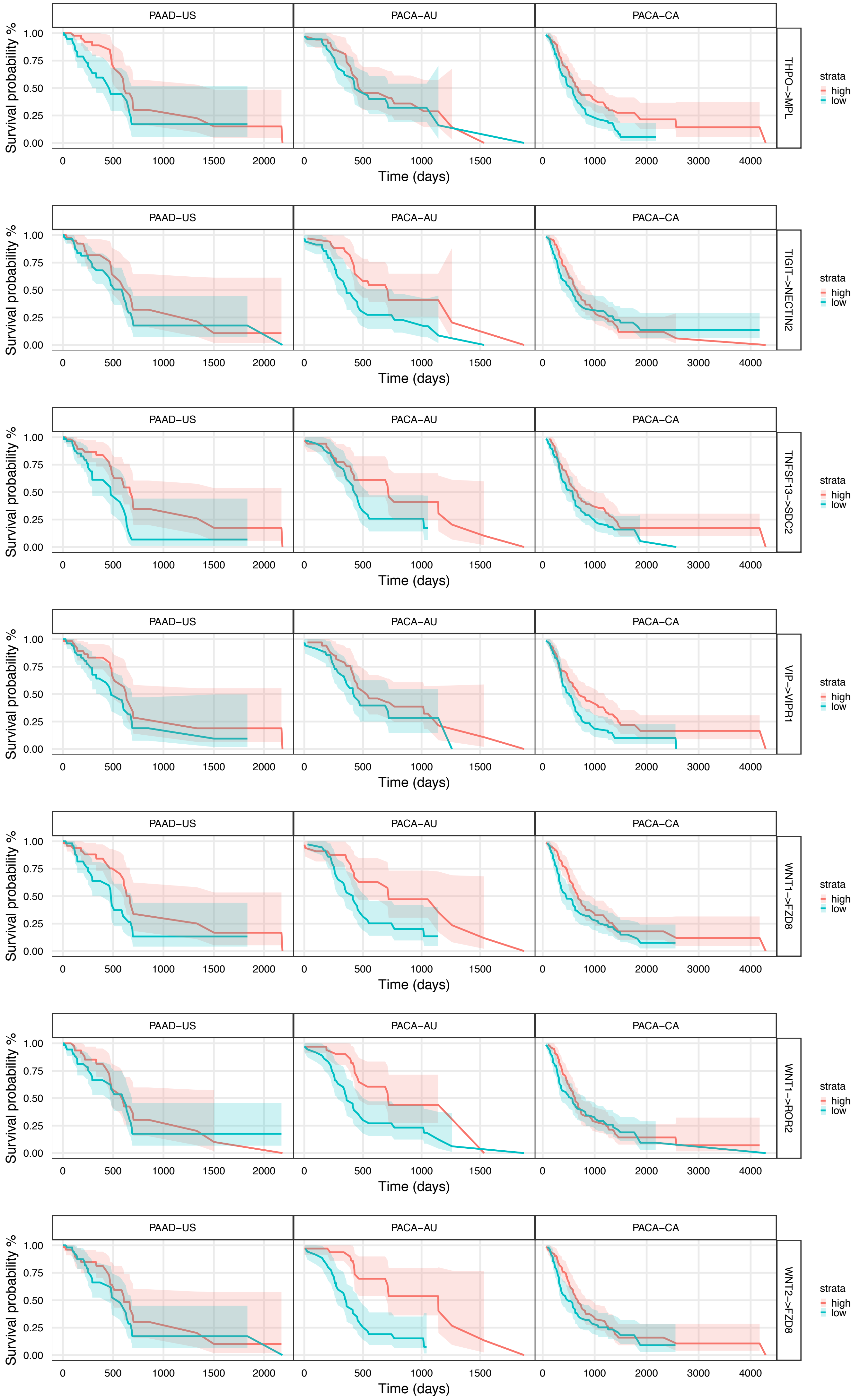
PAAD−US
PACA−AU
PACA−CA
Survival probability %
1.00
0.75
0.50
0.25
0.00
0
500
1000
1500
2000
3000
4000
Time (days)
THPO−>MPL
TIGIT−>NECTIN2
TNFSF13−>SDC2
VIP−>VIPR1
WNT1−>FZD8
WNT1−>ROR2
WNT2−>FZD8
strata
high
low

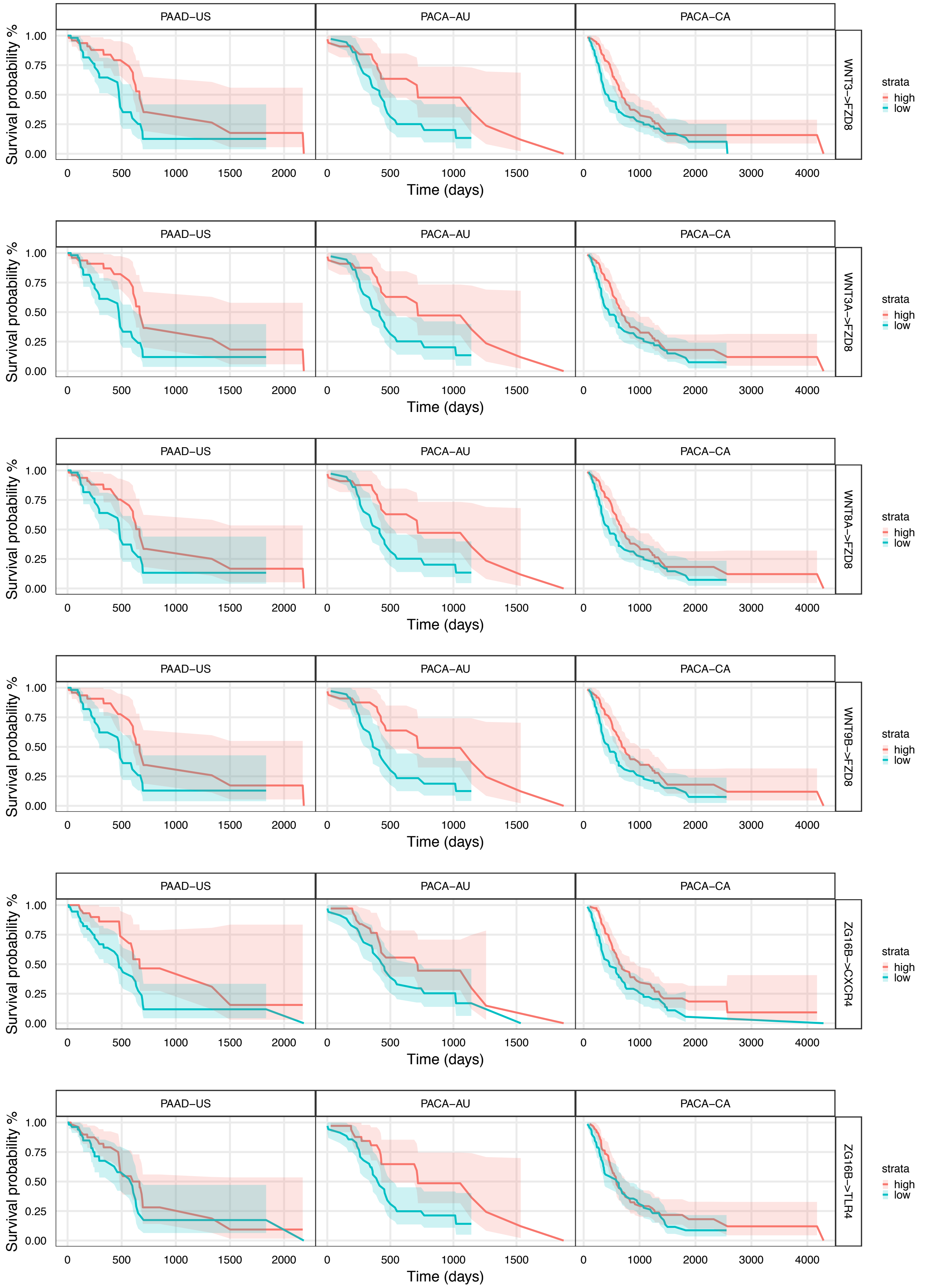

Figure S2. Kaplan-Meier plots of good-prognostic LR pairs.

Kaplan-Meier plots of good-prognostic LR pairs for each cohort. Hazard ratios, p-values, and q-values are shown in **Table S2**.

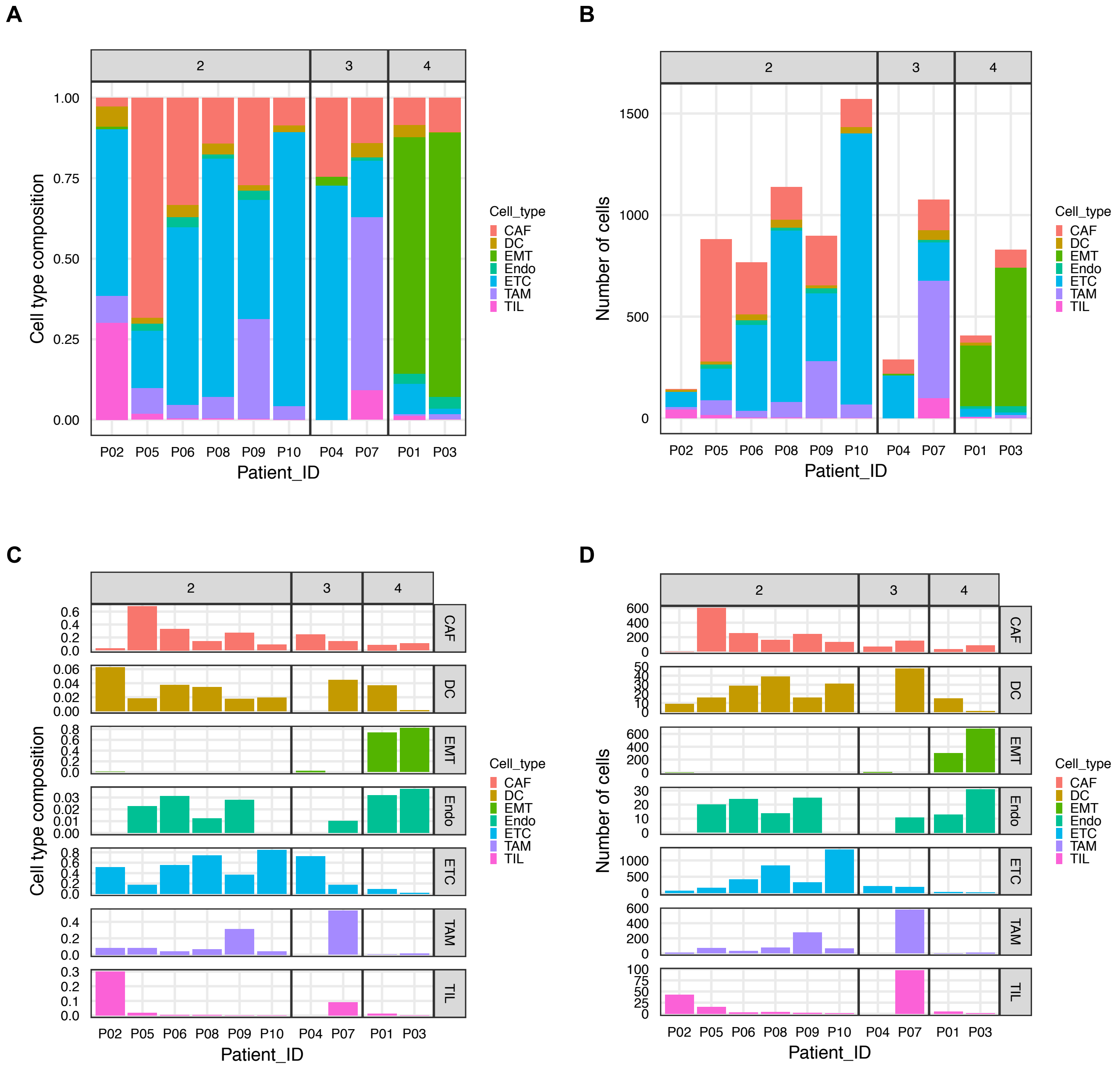

Figure S3. Cell-type compositions and numbers of cells in the scRNA-seq data.

(A) Cell-type composition per cell type and patient. The numbers on the top indicate patients' grades. (B) Numbers of cells per cell type and patient. The numbers on the top indicate patients' grades. (C) Cell-type composition per cell type and patient. The numbers on the top indicate patients' grades. (D) Numbers of cells per cell type and patient. The numbers on the top indicate patients' grades.

A

All LR pairs

(2278 LR pairs)

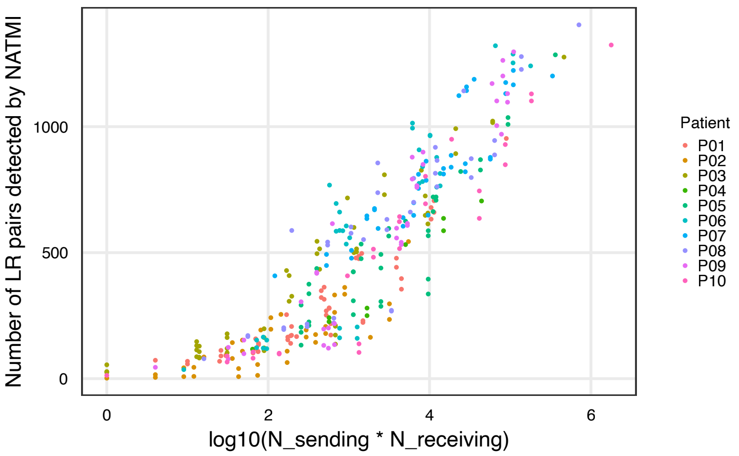

B

Poor-prognostic LR pairs

(67 LR pairs)

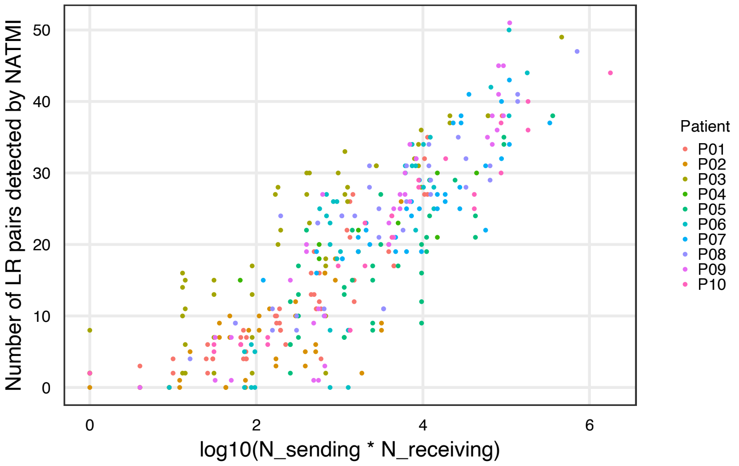

C

Good-prognostic LR pairs

(132 LR pairs)

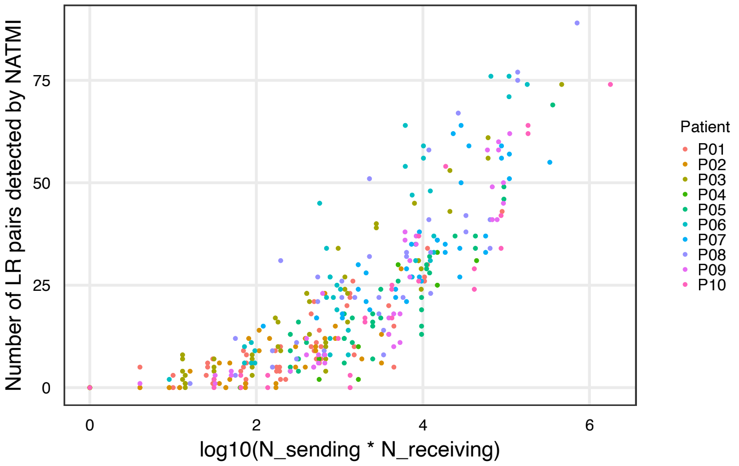

Figure S4. Relationship of the number of cells with the number of detected LR pairs per cell-type pairs. Relationship of the number of cells with the number of detected LR pairs per cell-type pair and patient for (A) all LR pairs, (B) poor-prognostic LR pairs, and (C) good-prognostic LR pairs. The x-axes represent the log-transformed product of the numbers of cells for two cell types. The y-axes represent the number of LR pairs detected by NATMI per patient. Point colors represent patients.

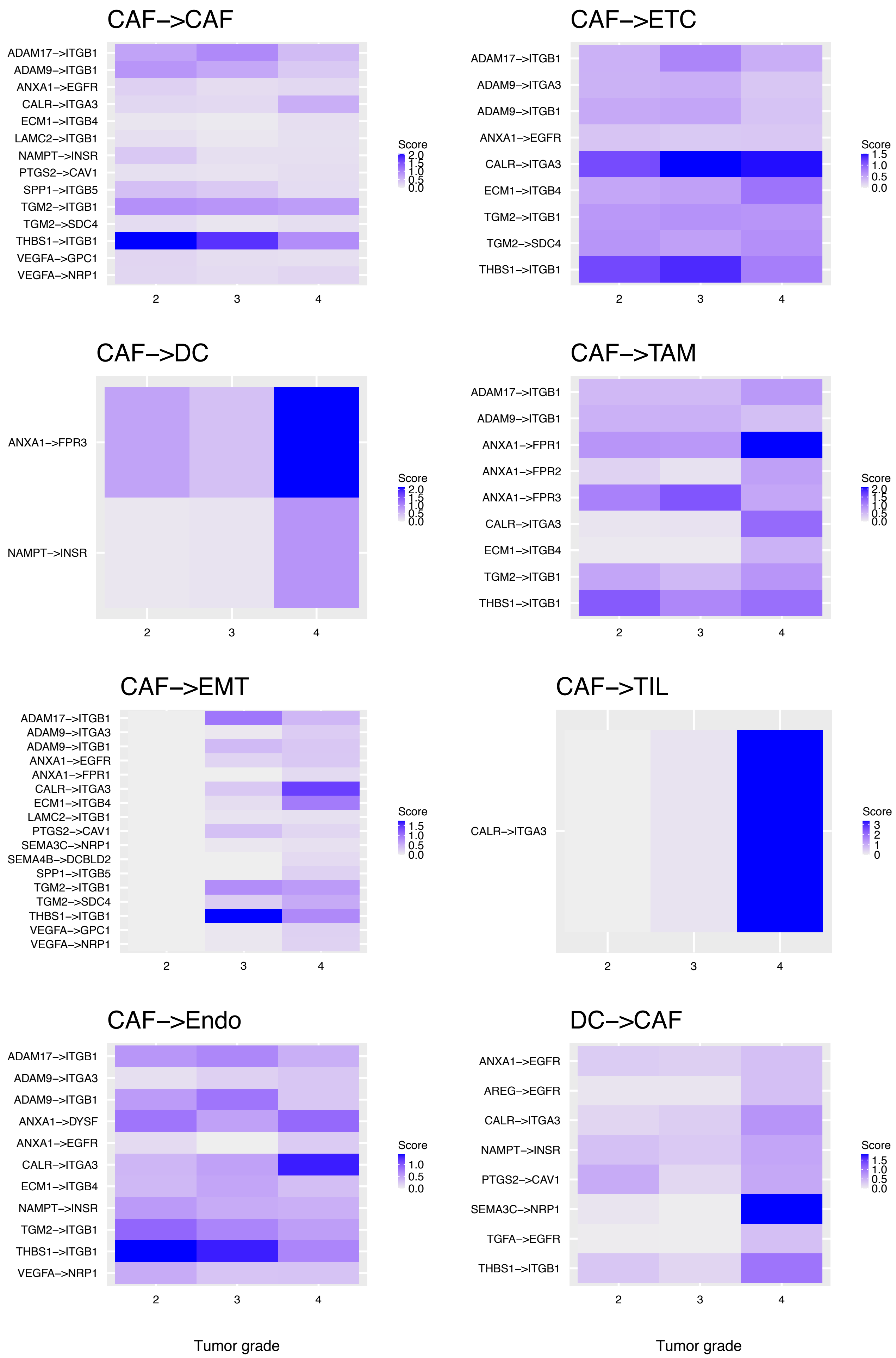
CAF–>CAF
ADAM17–>ITGB1
ADAM9–>ITGB1
ANXA1–>EGFR
CALR–>ITGA3
ECM1–>ITGB4
LAMC2–>ITGB1
NAMPT–>INSR
PTGS2–>CAV1
SPP1–>ITGB5
TGM2–>ITGB1
TGM2–>SDC4
THBS1–>ITGB1
VEGFA–>GPC1
VEGFA–>NRP1
Score
2.0
1.5
1.0
0.5
0.0
CAF–>ETC
ADAM17–>ITGB1
ADAM9–>ITGA3
ADAM9–>ITGB1
ANXA1–>EGFR
CALR–>ITGA3
ECM1–>ITGB4
TGM2–>ITGB1
TGM2–>SDC4
THBS1–>ITGB1
Score
1.5
1.0
0.5
0.0
CAF–>DC
ANXA1–>FPR3
NAMPT–>INSR
Score
2.0
1.5
1.0
0.5
0.0
CAF–>TAM
ADAM17–>ITGB1
ADAM9–>ITGB1
ANXA1–>FPR1
ANXA1–>FPR2
ANXA1–>FPR3
CALR–>ITGA3
ECM1–>ITGB4
TGM2–>ITGB1
THBS1–>ITGB1
Score
2.0
1.5
1.0
0.5
0.0
CAF–>EMT
ADAM17–>ITGB1
ADAM9–>ITGA3
ADAM9–>ITGB1
ANXA1–>EGFR
ANXA1–>FPR1
CALR–>ITGA3
ECM1–>ITGB4
LAMC2–>ITGB1
PTGS2–>CAV1
SEMA3C–>NRP1
SEMA4B–>DCBLD2
SPP1–>ITGB5
TGM2–>ITGB1
TGM2–>SDC4
THBS1–>ITGB1
VEGFA–>GPC1
VEGFA–>NRP1
Score
1.5
1.0
0.5
0.0
CAF–>TIL
CALR–>ITGA3
Score
3
2
1
0
CAF–>Endo
ADAM17–>ITGB1
ADAM9–>ITGA3
ADAM9–>ITGB1
ANXA1–>DYSF
ANXA1–>EGFR
CALR–>ITGA3
ECM1–>ITGB4
NAMPT–>INSR
TGM2–>ITGB1
THBS1–>ITGB1
VEGFA–>NRP1
Score
1.0
0.5
0.0
DC–>CAF
ANXA1–>EGFR
AREG–>EGFR
CALR–>ITGA3
NAMPT–>INSR
PTGS2–>CAV1
SEMA3C–>NRP1
TGFA–>EGFR
THBS1–>ITGB1
Score
1.5
1.0
0.5
0.0
2
3
4
Tumor grade

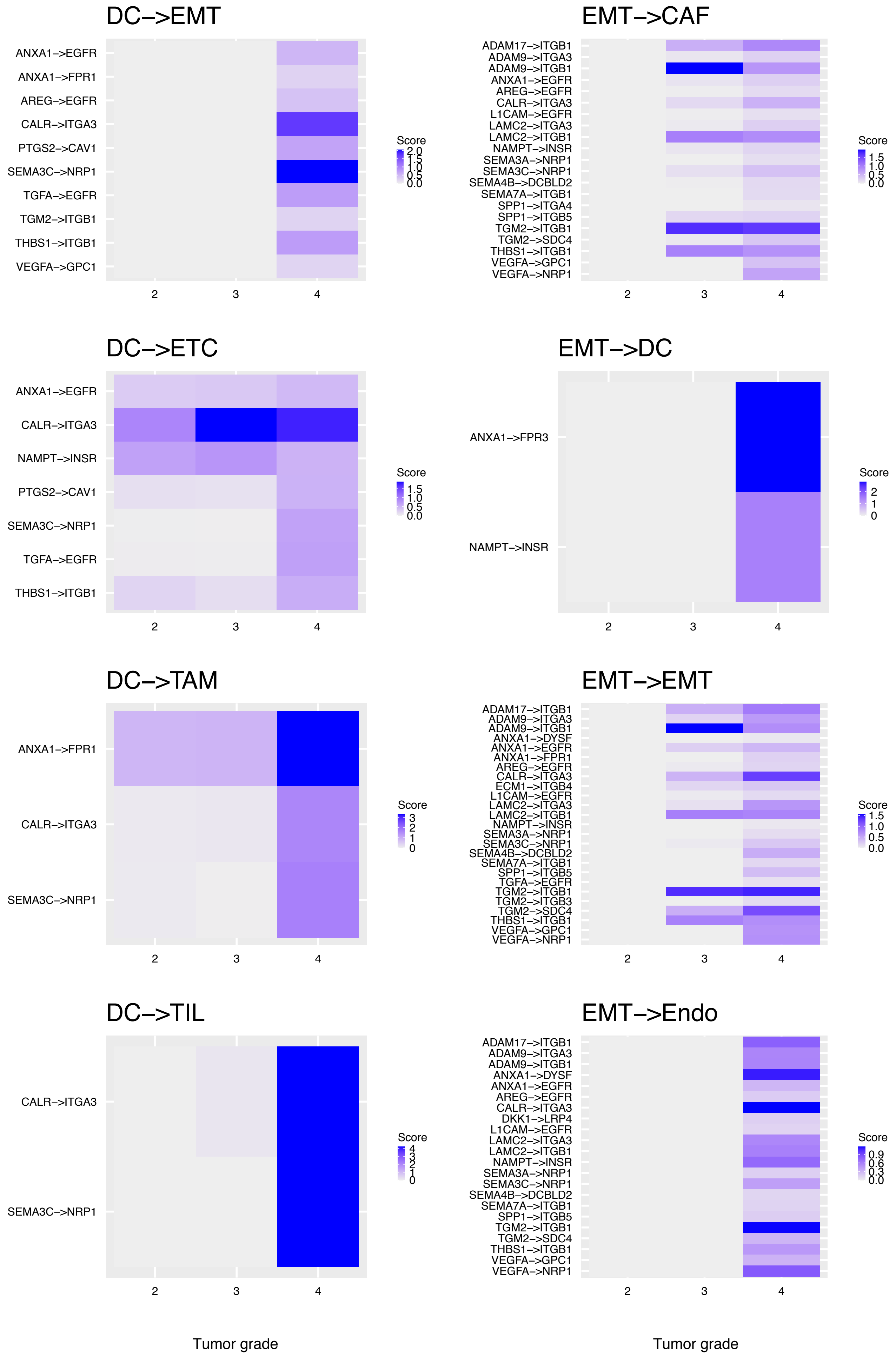
DC–>EMT
ANXA1–>EGFR
ANXA1–>FPR1
AREG–>EGFR
CALR–>ITGA3
PTGS2–>CAV1
SEMA3C–>NRP1
TGFA–>EGFR
TGM2–>ITGB1
THBS1–>ITGB1
VEGFA–>GPC1
Score
2.0
1.5
1.0
0.5
0.0
2
3
4
EMT–>CAF
ADAM17–>ITGB1
ADAM9–>ITGA3
ADAM9–>ITGB1
ANXA1–>EGFR
AREG–>EGFR
CALR–>ITGA3
L1CAM–>EGFR
LAMC2–>ITGA3
LAMC2–>ITGB1
NAMPT–>INSR
SEMA3A–>NRP1
SEMA3C–>NRP1
SEMA4B–>DCBLD2
SEMA7A–>ITGB1
SPP1–>ITGA4
SPP1–>ITGB5
TGM2–>ITGB1
TGM2–>SDC4
THBS1–>ITGB1
VEGFA–>GPC1
VEGFA–>NRP1
Score
1.5
1.0
0.5
0.0
2
3
4
DC–>ETC
ANXA1–>EGFR
CALR–>ITGA3
NAMPT–>INSR
PTGS2–>CAV1
SEMA3C–>NRP1
TGFA–>EGFR
THBS1–>ITGB1
Score
1.5
1.0
0.5
0.0
2
3
4
EMT–>DC
ANXA1–>FPR3
NAMPT–>INSR
Score
2
1
0
2
3
4
DC–>TAM
ANXA1–>FPR1
CALR–>ITGA3
SEMA3C–>NRP1
Score
3
2
1
0
2
3
4
EMT–>EMT
ADAM17–>ITGB1
ADAM9–>ITGA3
ADAM9–>ITGB1
ANXA1–>DYSF
ANXA1–>EGFR
ANXA1–>FPR1
AREG–>EGFR
CALR–>ITGA3
ECM1–>ITGB4
L1CAM–>EGFR
LAMC2–>ITGA3
LAMC2–>ITGB1
NAMPT–>INSR
SEMA3A–>NRP1
SEMA3C–>NRP1
SEMA4B–>DCBLD2
SEMA7A–>ITGB1
SPP1–>ITGB5
TGFA–>EGFR
TGM2–>ITGB1
TGM2–>ITGB3
TGM2–>SDC4
THBS1–>ITGB1
VEGFA–>GPC1
VEGFA–>NRP1
Score
1.5
1.0
0.5
0.0
2
3
4
DC–>TIL
CALR–>ITGA3
SEMA3C–>NRP1
Score
4
3
2
1
0
2
3
4
EMT–>Endo
ADAM17–>ITGB1
ADAM9–>ITGA3
ADAM9–>ITGB1
ANXA1–>DYSF
ANXA1–>EGFR
AREG–>EGFR
CALR–>ITGA3
DKK1–>LRP4
L1CAM–>EGFR
LAMC2–>ITGA3
LAMC2–>ITGB1
NAMPT–>INSR
SEMA3A–>NRP1
SEMA3C–>NRP1
SEMA4B–>DCBLD2
SEMA7A–>ITGB1
SPP1–>ITGB5
TGM2–>ITGB1
TGM2–>SDC4
THBS1–>ITGB1
VEGFA–>GPC1
VEGFA–>NRP1
Score
0.9
0.6
0.3
0.0
2
3
4
Tumor grade
Tumor grade

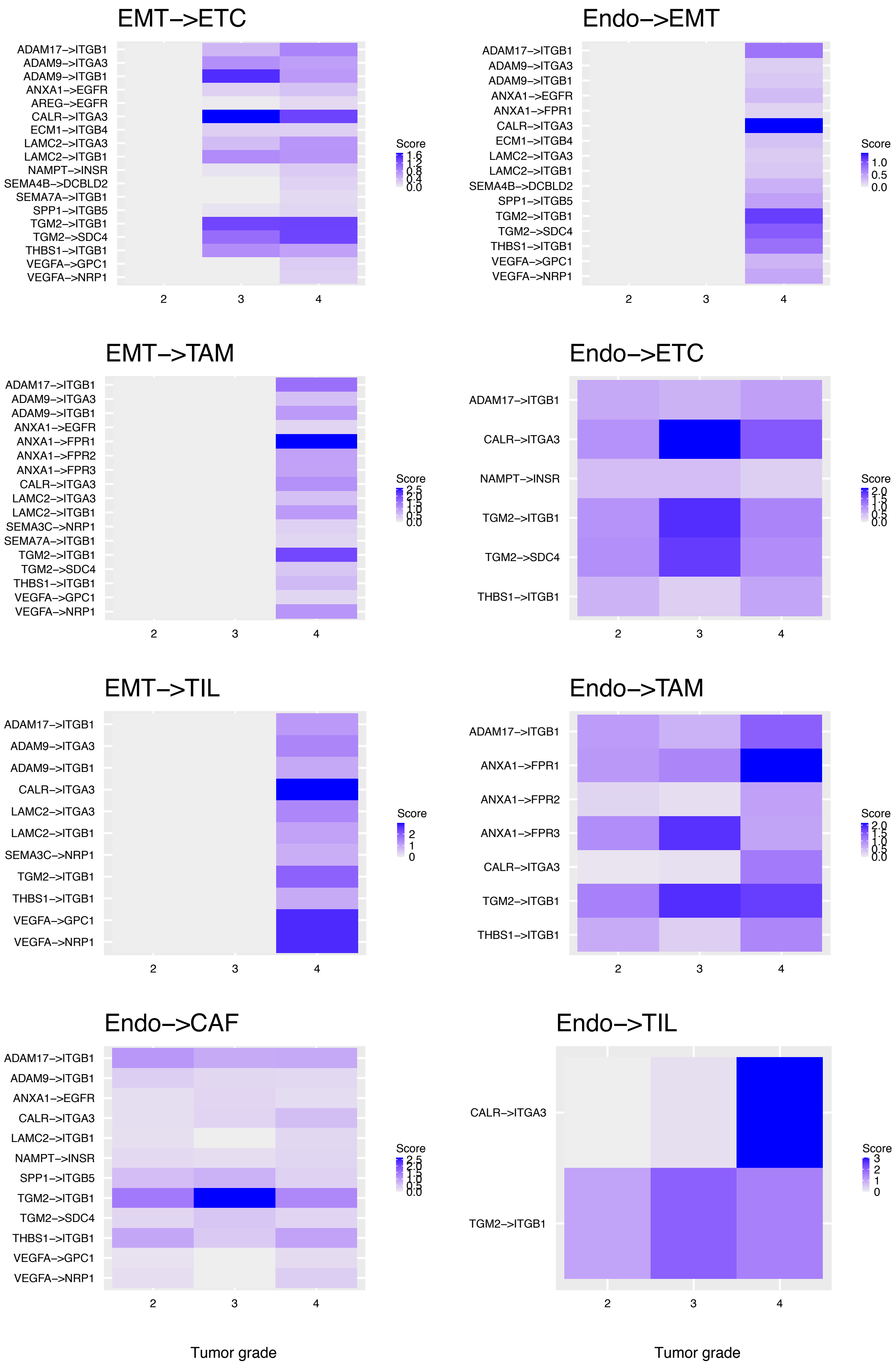
EMT–>ETC
ADAM17–>ITGB1
ADAM9–>ITGA3
ADAM9–>ITGB1
ANXA1–>EGFR
AREG–>EGFR
CALR–>ITGA3
ECM1–>ITGB4
LAMC2–>ITGA3
LAMC2–>ITGB1
NAMPT–>INSR
SEMA4B–>DCBLD2
SEMA7A–>ITGB1
SPP1–>ITGB5
TGM2–>ITGB1
TGM2–>SDC4
THBS1–>ITGB1
VEGFA–>GPC1
VEGFA–>NRP1
2
3
4
Score
1.6
1.2
0.8
0.4
0.0
Endo–>EMT
ADAM17–>ITGB1
ADAM9–>ITGA3
ADAM9–>ITGB1
ANXA1–>EGFR
ANXA1–>FPR1
CALR–>ITGA3
ECM1–>ITGB4
LAMC2–>ITGA3
LAMC2–>ITGB1
SEMA4B–>DCBLD2
SPP1–>ITGB5
TGM2–>ITGB1
TGM2–>SDC4
THBS1–>ITGB1
VEGFA–>GPC1
VEGFA–>NRP1
2
3
4
Score
1.0
0.5
0.0
EMT–>TAM
ADAM17–>ITGB1
ADAM9–>ITGA3
ADAM9–>ITGB1
ANXA1–>EGFR
ANXA1–>FPR1
ANXA1–>FPR2
ANXA1–>FPR3
CALR–>ITGA3
LAMC2–>ITGA3
LAMC2–>ITGB1
SEMA3C–>NRP1
SEMA7A–>ITGB1
TGM2–>ITGB1
TGM2–>SDC4
THBS1–>ITGB1
VEGFA–>GPC1
VEGFA–>NRP1
2
3
4
Score
2.5
2.0
1.5
1.0
0.5
0.0
Endo–>ETC
ADAM17–>ITGB1
CALR–>ITGA3
NAMPT–>INSR
TGM2–>ITGB1
TGM2–>SDC4
THBS1–>ITGB1
2
3
4
Score
2.0
1.5
1.0
0.5
0.0
EMT–>TIL
ADAM17–>ITGB1
ADAM9–>ITGA3
ADAM9–>ITGB1
CALR–>ITGA3
LAMC2–>ITGA3
LAMC2–>ITGB1
SEMA3C–>NRP1
TGM2–>ITGB1
THBS1–>ITGB1
VEGFA–>GPC1
VEGFA–>NRP1
2
3
4
Score
2
1
0
Endo–>TAM
ADAM17–>ITGB1
ANXA1–>FPR1
ANXA1–>FPR2
ANXA1–>FPR3
CALR–>ITGA3
TGM2–>ITGB1
THBS1–>ITGB1
2
3
4
Score
2.0
1.5
1.0
0.5
0.0
Endo–>CAF
ADAM17–>ITGB1
ADAM9–>ITGB1
ANXA1–>EGFR
CALR–>ITGA3
LAMC2–>ITGB1
NAMPT–>INSR
SPP1–>ITGB5
TGM2–>ITGB1
TGM2–>SDC4
THBS1–>ITGB1
VEGFA–>GPC1
VEGFA–>NRP1
2
3
4
Score
2.5
2.0
1.5
1.0
0.5
0.0
Endo–>TIL
CALR–>ITGA3
TGM2–>ITGB1
2
3
4
Score
3
2
1
0
Tumor grade
Tumor grade

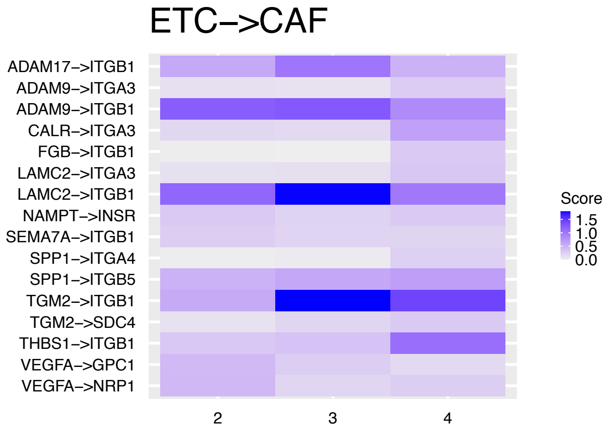
ETC–>CAF
ADAM17–>ITGB1
ADAM9–>ITGA3
ADAM9–>ITGB1
CALR–>ITGA3
FGB–>ITGB1
LAMC2–>ITGA3
LAMC2–>ITGB1
NAMPT–>INSR
SEMA7A–>ITGB1
SPP1–>ITGA4
SPP1–>ITGB5
TGM2–>ITGB1
TGM2–>SDC4
THBS1–>ITGB1
VEGFA–>GPC1
VEGFA–>NRP1
2
3
4
Score
1.5
1.0
0.5
0.0

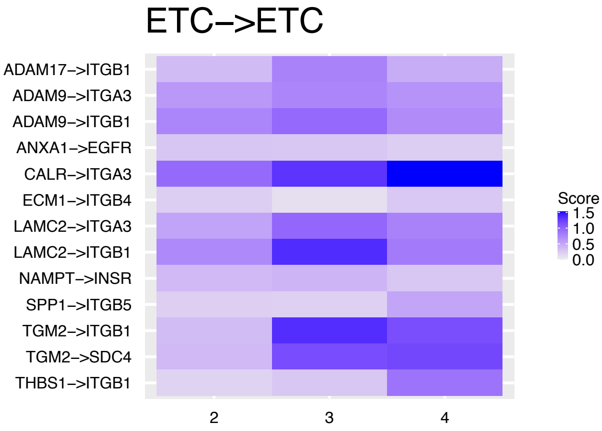
ETC–>ETC
ADAM17–>ITGB1
ADAM9–>ITGA3
ADAM9–>ITGB1
ANXA1–>EGFR
CALR–>ITGA3
ECM1–>ITGB4
LAMC2–>ITGA3
LAMC2–>ITGB1
NAMPT–>INSR
SPP1–>ITGB5
TGM2–>ITGB1
TGM2–>SDC4
THBS1–>ITGB1
2
3
4
Score
1.5
1.0
0.5
0.0

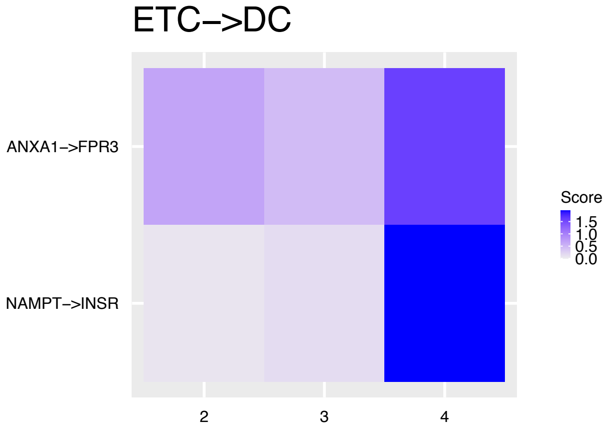
ETC–>DC
ANXA1–>FPR3
NAMPT–>INSR
2
3
4
Score
1.5
1.0
0.5
0.0

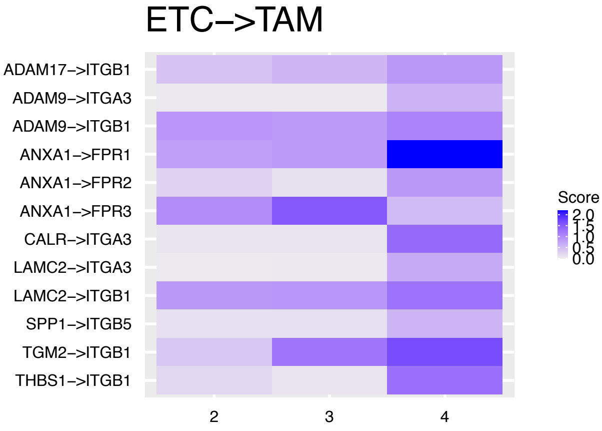
ETC–>TAM
ADAM17–>ITGB1
ADAM9–>ITGA3
ADAM9–>ITGB1
ANXA1–>FPR1
ANXA1–>FPR2
ANXA1–>FPR3
CALR–>ITGA3
LAMC2–>ITGA3
LAMC2–>ITGB1
SPP1–>ITGB5
TGM2–>ITGB1
THBS1–>ITGB1
2
3
4
Score
2.0
1.5
1.0
0.5
0.0

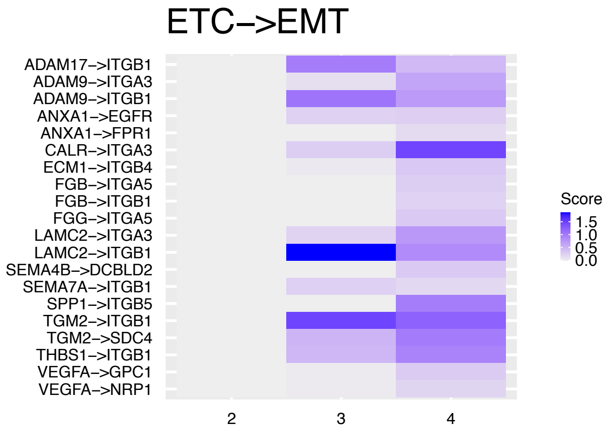
ETC–>EMT
ADAM17–>ITGB1
ADAM9–>ITGA3
ADAM9–>ITGB1
ANXA1–>EGFR
ANXA1–>FPR1
CALR–>ITGA3
ECM1–>ITGB4
FGB–>ITGA5
FGB–>ITGB1
FGG–>ITGA5
LAMC2–>ITGA3
LAMC2–>ITGB1
SEMA4B–>DCBLD2
SEMA7A–>ITGB1
SPP1–>ITGB5
TGM2–>ITGB1
TGM2–>SDC4
THBS1–>ITGB1
VEGFA–>GPC1
VEGFA–>NRP1
2
3
4
Score
1.5
1.0
0.5
0.0

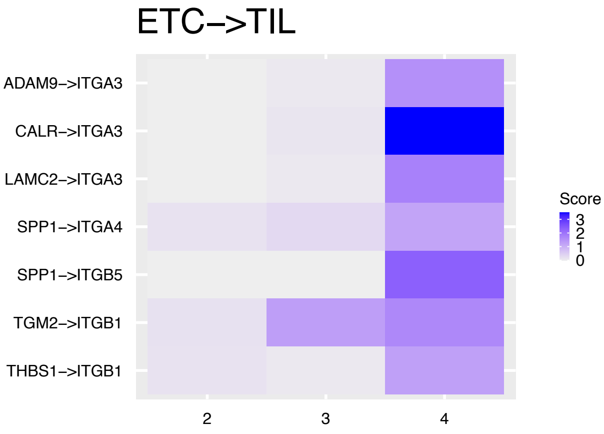
ETC–>TIL
ADAM9–>ITGA3
CALR–>ITGA3
LAMC2–>ITGA3
SPP1–>ITGA4
SPP1–>ITGB5
TGM2–>ITGB1
THBS1–>ITGB1
2
3
4
Score
3
2
1
0

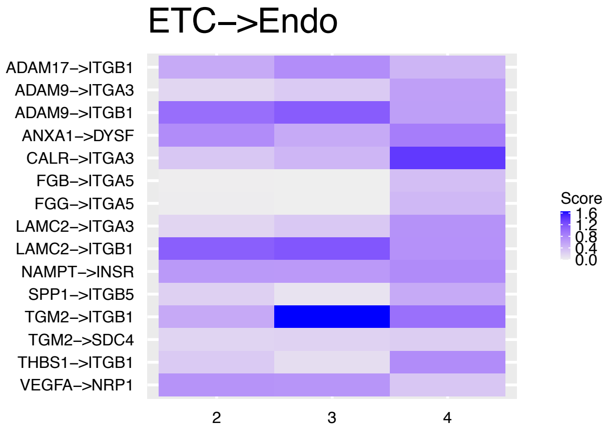
ETC–>Endo
ADAM17–>ITGB1
ADAM9–>ITGA3
ADAM9–>ITGB1
ANXA1–>DYSF
CALR–>ITGA3
FGB–>ITGA5
FGG–>ITGA5
LAMC2–>ITGA3
LAMC2–>ITGB1
NAMPT–>INSR
SPP1–>ITGB5
TGM2–>ITGB1
TGM2–>SDC4
THBS1–>ITGB1
VEGFA–>NRP1
2
3
4
Score
1.6
1.2
0.8
0.4
0.0

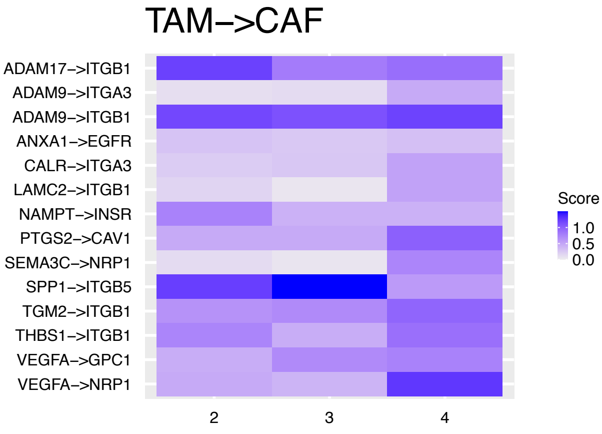
TAM–>CAF
ADAM17–>ITGB1
ADAM9–>ITGA3
ADAM9–>ITGB1
ANXA1–>EGFR
CALR–>ITGA3
LAMC2–>ITGB1
NAMPT–>INSR
PTGS2–>CAV1
SEMA3C–>NRP1
SPP1–>ITGB5
TGM2–>ITGB1
THBS1–>ITGB1
VEGFA–>GPC1
VEGFA–>NRP1
2
3
4
Score
1.0
0.5
0.0

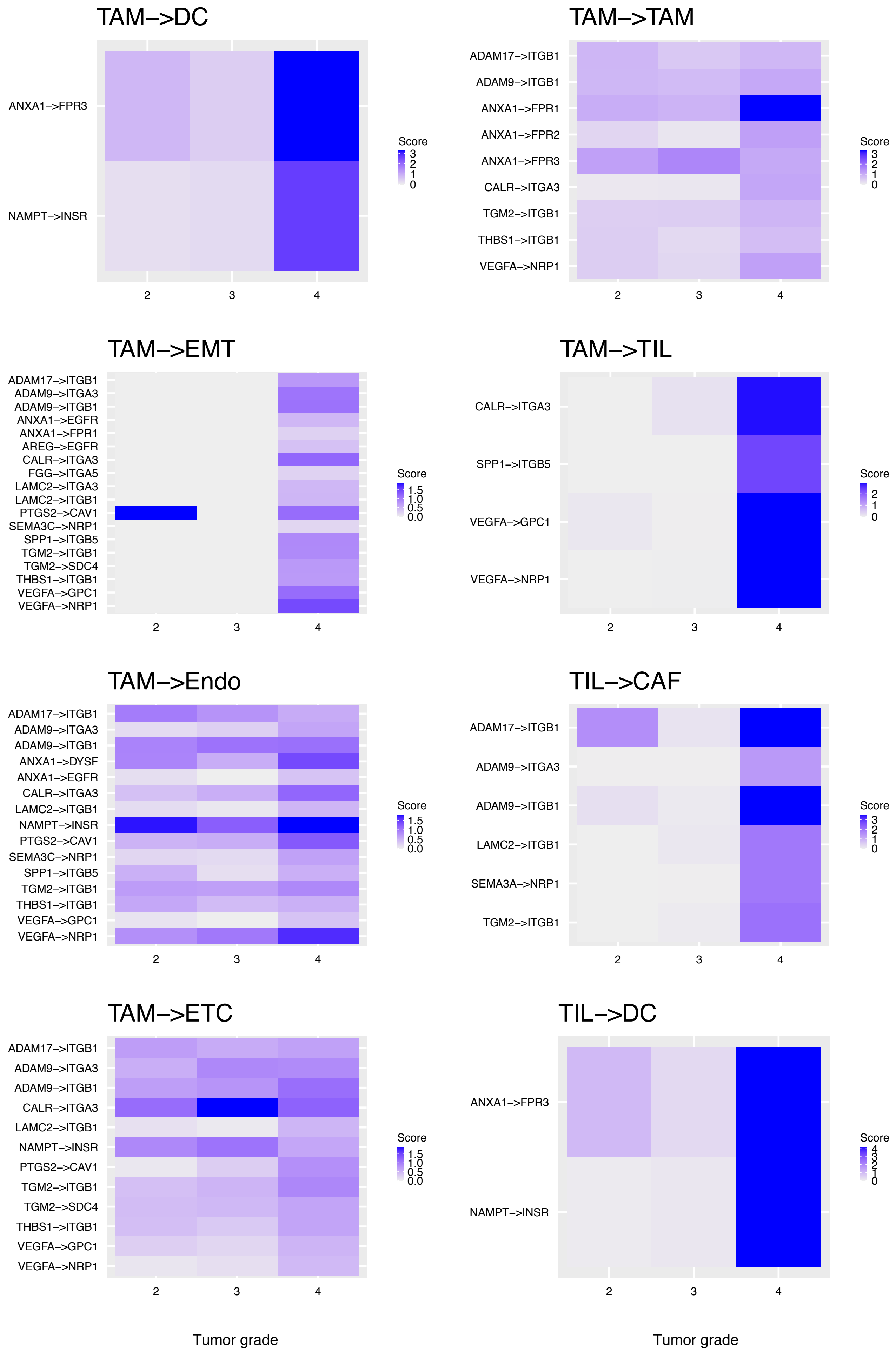
TAM–>DC
ANXA1–>FPR3
NAMPT–>INSR
2 3 4
Score 3 2 1 0
TAM–>TAM
ADAM17–>ITGB1
ADAM9–>ITGB1
ANXA1–>FPR1
ANXA1–>FPR2
ANXA1–>FPR3
CALR–>ITGA3
TGM2–>ITGB1
THBS1–>ITGB1
VEGFA–>NRP1
2 3 4
Score 3 2 1 0
TAM–>EMT
ADAM17–>ITGB1
ADAM9–>ITGA3
ADAM9–>ITGB1
ANXA1–>EGFR
ANXA1–>FPR1
AREG–>EGFR
CALR–>ITGA3
FGG–>ITGA5
LAMC2–>ITGA3
LAMC2–>ITGB1
PTGS2–>CAV1
SEMA3C–>NRP1
SPP1–>ITGB5
TGM2–>ITGB1
TGM2–>SDC4
THBS1–>ITGB1
VEGFA–>GPC1
VEGFA–>NRP1
2 3 4
Score 1.5 1.0 0.5 0.0
TAM–>TIL
CALR–>ITGA3
SPP1–>ITGB5
VEGFA–>GPC1
VEGFA–>NRP1
2 3 4
Score 2 1 0
TAM–>Endo
ADAM17–>ITGB1
ADAM9–>ITGA3
ADAM9–>ITGB1
ANXA1–>DYSF
ANXA1–>EGFR
CALR–>ITGA3
LAMC2–>ITGB1
NAMPT–>INSR
PTGS2–>CAV1
SEMA3C–>NRP1
SPP1–>ITGB5
TGM2–>ITGB1
THBS1–>ITGB1
VEGFA–>GPC1
VEGFA–>NRP1
2 3 4
Score 1.5 1.0 0.5 0.0
TIL–>CAF
ADAM17–>ITGB1
ADAM9–>ITGA3
ADAM9–>ITGB1
LAMC2–>ITGB1
SEMA3A–>NRP1
TGM2–>ITGB1
2 3 4
Score 3 2 1 0
TAM–>ETC
ADAM17–>ITGB1
ADAM9–>ITGA3
ADAM9–>ITGB1
CALR–>ITGA3
LAMC2–>ITGB1
NAMPT–>INSR
PTGS2–>CAV1
TGM2–>ITGB1
TGM2–>SDC4
THBS1–>ITGB1
VEGFA–>GPC1
VEGFA–>NRP1
2 3 4
Score 1.5 1.0 0.5 0.0
TIL–>DC
ANXA1–>FPR3
NAMPT–>INSR
2 3 4
Score 4 3 2 1 0
Tumor grade
Tumor grade

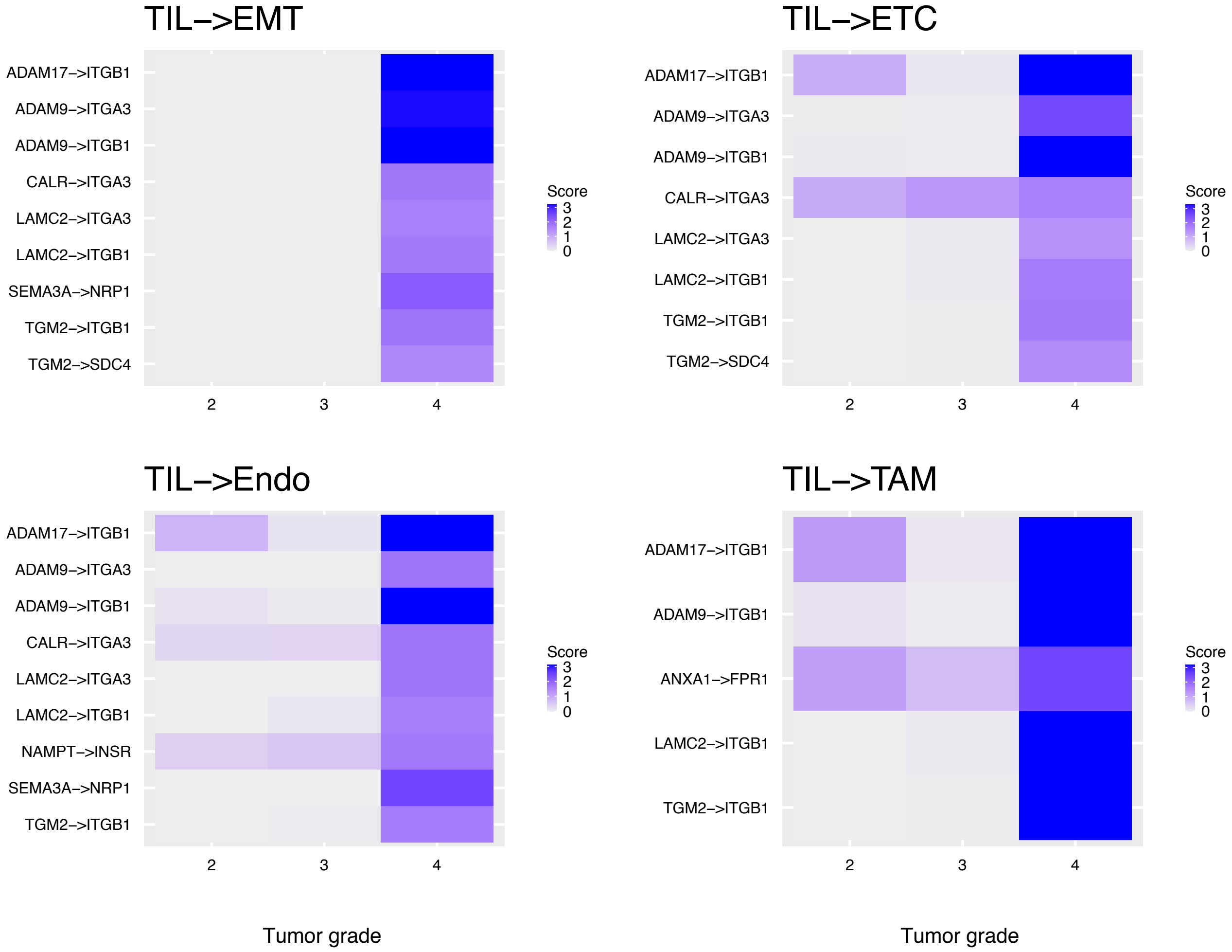

Figure S5. Heatmaps of mean expression weights in 33 grade-dependent LR pairs.

To screen CCIs related to a high mean expression weight at pathological grade 4 in the patients, we calculated the quartiles of the weights of all LR pairs detected in grade 4. Then, we selected LR pairs with the weights above the third quartile. “Score” represents mean expression weight.
